# Connecting Variability in Global Transcription Rate to Mitochondrial Variability

**DOI:** 10.1371/journal.pbio.1000560

**Published:** 2010-12-14

**Authors:** Ricardo Pires das Neves, Nick S. Jones, Lorena Andreu, Rajeev Gupta, Tariq Enver, Francisco J. Iborra

**Affiliations:** 1 Medical Research Council Molecular Haematology Unit, Weatherall Institute of Molecular Medicine, John Radcliffe Hospital, Oxford, United Kingdom; 2 Biocant Center of Innovation and Biotechnology, Cantanhede, Portugal; 3 Center for Neuroscience and Cell Biology University of Coimbra, Coimbra, Portugal; 4 Department of Physics and Biochemistry, Oxford Centre for Integrative Systems Biology, CABDyN Complexity Centre, Oxford, United Kingdom; 5 Department of Molecular and Cellular Biology, Centro Nacional de Biotecnología, Consejo Superior de Investigaciones Científicas, Madrid, Spain; University of California San Francisco/Howard Hughes Medical Institute, United States of America

## Abstract

Populations of genetically identical eukaryotic cells show significant cell-to-cell variability in gene expression. However, we lack a good understanding of the origins of this variation. We have found marked cell-to-cell variability in average cellular rates of transcription. We also found marked cell-to-cell variability in the amount of cellular mitochondrial mass. We undertook fusion studies that suggested that variability in transcription rate depends on small diffusible factors. Following this, in vitro studies showed that transcription rate has a sensitive dependence on [ATP] but not on the concentration of other nucleotide triphosphates (NTPs). Further experiments that perturbed populations by changing nutrient levels and available [ATP] suggested this connection holds in vivo. We found evidence that cells with higher mitochondrial mass, or higher total membrane potential, have a faster rate of transcription per unit volume of nuclear material. We also found evidence that transcription rate variability is substantially modulated by the presence of anti- or prooxidants. Daughter studies showed that a cause of variability in mitochondrial content is apparently stochastic segregation of mitochondria at division. We conclude by noting that daughters that stochastically inherit a lower mitochondrial mass than their sisters have relatively longer cell cycles. Our findings reveal a link between variability in energy metabolism and variability in transcription rate.

## Abstract 2

The authors demonstrate a connection between variability in the rate of transcription and differences in cellular mitochondrial content.

## Author Summary

Though pairs of cells may have identical genes, they still show behavioural differences. These cell-to-cell differences may arise from variations in how genes are transcribed and translated by the cellular machinery. Identifying the origins of this variation is important as it helps us understand why genetically identical cells can show a range of responses to the environment. In this work, we measured the rate at which the genes yield transcripts in cultured human cells. We found marked cell-to-cell variability in average rates of transcription. This variability is related to mitochondrial content. Cells with a higher mitochondrial mass have a faster rate of transcription, and we show that part of this variability is due to the unequal distribution of mitochondria to daughter cells when cells divide. Additionally, we find that cells that inherit more mitochondria divide earlier. These findings make a connection between variability in transcript production and variability in cellular mitochondrial content.

## Introduction

Genetically identical populations of cells can exhibit cell-to-cell variations in the amount of individual gene products; this can result in phenotypic diversity [1],[2]. The study of cellular variability was pioneered by Delbrück in the mid-forties, who measured differences in the number of phages produced by individual *Escherichia coli* [3]. Fluctuations in the small numbers of molecules involved in gene expression have been indicated as a source of this variation, and current experimental and theoretical approaches seek to anatomize the potential sources of variability, or “noise”. Variation between cells could be due to global factors such as cell cycle position or differences in numbers of transcription factors. Such changes can affect all genes and so constitute “extrinsic” sources of variability. In contrast, “intrinsic” noise is identified as molecular variation that occurs at the level of single genes and their products [4]. Cell-to-cell variability could be mainly the combined effect of large amounts of intrinsic variation or might be attributable to more system-wide extrinsic variation. In the following we investigate how global factors can influence transcription rate across the eukaryotic cell.

Experiments investigating gene expression noise suggest that gene expression variability has a mix of intrinsic and extrinsic sources [5],[6]. Intrinsic noise has been modelled extensively, and we have a relatively refined idea of its origin in the molecular machinery of transcription, translation, and degradation [1],[2],[7],[8]. The magnitude of extrinsic noise is largest at intermediate levels of gene expression and dominates when gene expression is high [6],[7],[9]. However, the sources of extrinsic noise are not as well characterised as those of intrinsic noise [7],[10]. Studies carried out in yeast have, for example, suggested cell size, cell shape, cell cycle stage, and fluctuations in an as yet unidentified upstream regulator as potential sources of extrinsic noise [9],[11]–[13]. While there has been discussion of variability in the process of transcription both in polymerase binding and in transcription elongation, e.g., [14]–[16], this is often with the principal aim of understanding intrinsic noise: in the following we will investigate how extrinsic factors might modulate transcription rate.

To investigate the origins of global variability in eukaryotic gene expression we undertook a study of global transcription rate. We define global transcription rate as the average rate of production of transcripts within the nucleus of a single cell. Our results, obtained using direct measurement approaches, demonstrate that there is marked cell-to-cell variability in global transcription rate. The elongation rate of RNA polymerase II (RNA pol II) is a likely determinant of transcriptional rate, and we demonstrate that RNA pol II elongation is very sensitive to ATP concentration. We find that differences in [ATP] between cells relate to the transcription rate variability observed. We further find that the amount of mitochondrial mass and total membrane potential (indicated by total cellular luminescence of CMXRos dye) both correlate with transcription rate. Finally, we find that there is pronounced variability in mitochondrial mass in cellular populations and that a source of this variability is asymmetric segregation of mitochondria during mitosis. In combination these findings suggest that variability in mitochondrial content represents a likely source of global variability in transcription rate in eukaryotic cells.

## Results/Discussion

### Evidence for Global Variability in Transcription Rate

**Figure 1:**
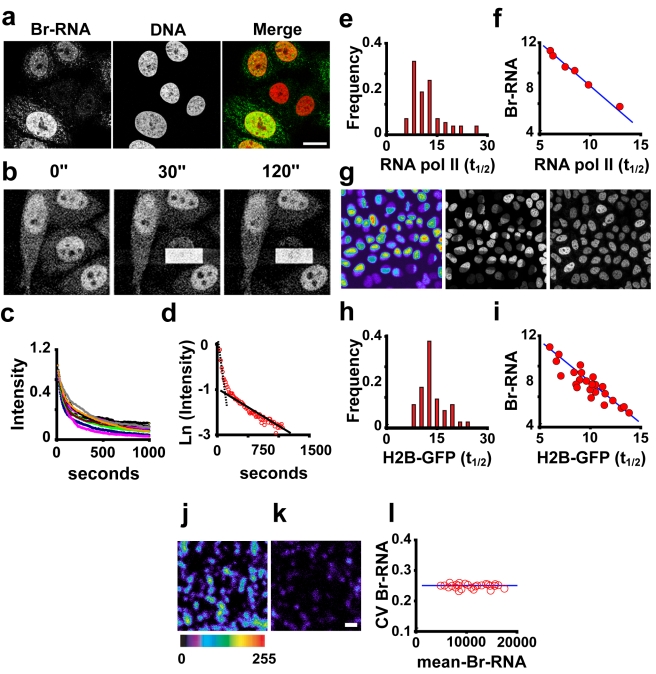
Global variability in transcription. (A) Variation in BrU incorporation. Hela cells were incubated for 30 min with 5 mM BrU. Br-RNA signal shows a large variation between cells (confocal section). (B). Kinetics of RNA pol II in vivo. FLIP analysis of GFP–RNA pol II. Half of the nucleus was bleached continuously as confocal images were collected approximately every 5 s. (C) Decay in fluorescence intensity of the unbleached area of the nucleus in the FLIP experiment (intensity, arbitrary units) (*n* = 60). (D) Log plot analysis of FLIP data showing two populations of RNA pol II molecules, as described in Hieda et al [23]. (E) Distribution of *t*_1/2_ for the DNA-associated form of RNA pol II in the cell population (*n* = 60). (F) BrU incorporation is correlated with the half-life of the DNA-associated RNA pol II population. After FLIP, cells were incubated with BrU, fixed, and immunolabelled for Br-RNA. Individual cells were identified, and the values of *t*_1/2_ for the DNA-associated form of RNA pol II and the corresponding Br-RNA intensity were plotted. (G–I) Histone H2B–GFP fast-exchanging population is a reporter of transcription elongation. (G) Hela cells expressing histone H2B–GFP were used for FLIP analysis (left panel). These cells were subsequently subjected to BrU incorporation. After identification of the same cells (middle panel), we measured Br-RNA production (right panel), which shows a good correlation (I). In (H) we show the variability in *t*_1/2_ for the rapid-exchangeable histone H2B–GFP (transcription-dependent fraction, Figure S4) (*n* = 100). Again, this shows a high variability between cells in a population. (J and K) RNA pol II molecules track at similar speed inside a given cell. High-power images of Br-RNA foci in two nuclei with different mean intensity of Br-RNA after 15 min of incorporation of BrU. The images have been pseudo-coloured to improve visualisation (purple = low, red = high). All the foci in an individual nucleus show a consistent level of Br-RNA intensity. (L) Analysis of the noise in Br-RNA production in transcription foci in individual cells. The plot shows 40 cells (>200 foci were analysed per cell). Transcription rates of Br-RNA foci appear to be correlated. Bars: (A), 10 µm; (K), 0.200 µm.

We directly measured transcription rate by recording levels of bromouridine (BrU) incorporated into nascent RNA [17],[18]. The intensity of the BrU signal in RNA containing BrU (Br-RNA) rises with time, reaching a plateau after 1 h of incubation (Figure S1), when equilibrium between synthesis and degradation is reached. In these experiments BrU levels were analysed on confocal sections, providing a measure of the transcription rate per unit of nuclear volume. After a short pulse of BrU (30 min) the amount of Br-RNA produced by different cells (Hela) varied dramatically across the cell population (Figures 1A and S1A). This variation in BrU incorporation per unit of nuclear volume was not limited to Hela cells, but was observed in other established mammalian cell lines (murine erythroleukemia cells and Chinese hamster ovary cells), immortalised cultures (EBV-transformed lymphoblasts and mouse embryonic stem cells), and, importantly, in primary cells (lymphocytes and primary human fibroblasts) with coefficients of variation (CVs) ranging from 0.3 to 0.6 (data not shown; CV is the standard deviation of the data points divided by their mean).

Many factors could possibly contribute to variability in BrU incorporation. One source of variation could be differences in staging between cells in a population [9],[12]. We observed that the variability in total nuclear BrU incorporation remains substantial throughout the cell cycle, from a CV of 0.36 in G1 to 0.35 in G2 (Figure S1D and S1E), and thus, in agreement with previous studies performed in yeast [11], the cell cycle was ruled out as a principal source of variability in BrU incorporation.

Another source of variability in BrU incorporation could be differences in the number of active molecules of RNA pol II between individual cells. Therefore, we estimated the number of active RNA pol II molecules in different cell types, using run-on experiments [18]–[20] (see Figure S2A and S2B). The results suggest that the amount of active RNA pol II molecules was approximately constant per unit of nuclear volume in a given population and across the different cell types analysed. This suggests that the variation observed in BrU incorporation is not due to differences in the number of active RNA pol II molecules between individual cells.

We next asked whether variability in transcription rate by RNA pol II could account for the differences in BrU incorporation observed. The transcription cycle by RNA pol II can be understood as follows: free RNA pol II molecules interact with DNA, making a complex that can either be abortive (binding to DNA and not transcribing, or transcribing a very short transcript) or that can proceed into elongation mode after being modified. Once RNA pol II elongating molecules finish the transcription cycle, they become free and diffuse throughout the nucleoplasm. This simple model thus involves steps with different kinetic properties, which we exploited to gain insight into the rate of transcription of RNA pol II in single cells. We generated a cell line (C23) in which a GFP-tagged version of wild-type RNA pol II was introduced into Chinese hamster ovary cells containing a temperature-sensitive mutation in the largest catalytic subunit of RNA pol II (tsTM4). At the restrictive temperature, only the wild-type GFP–RNA pol II was functional [21], complementing the endogenous RNA pol II mutant (tsTM4) and thereby enabling the mutant Chinese hamster ovary cells to grow normally [22]. We performed fluorescence loss in photobleaching (FLIP) analysis of the wild-type GFP–RNA pol II (Figure 1B–1D) and obtained *K*_off_ values for RNA pol II consistent with the presence of at least two populations of RNA pol II molecules (Figures 1D and S3), as has been previously suggested: one freely diffusible (short half-life), and another associated with the DNA (long half-life) [21],[23]. When we analysed the *K*_off_ data from individual cells of the DNA-associated population (long half-life), we found a huge variation between cells (Figure 1E), with a half-life (*t*_1/2_) ranging from 2.5 to 30 min with a mean of 10.1 min and a standard deviation of 4.9 min, suggesting the existence of significant variation in rates of transcription elongation. The FLIP analysis exhibited a CV of 0.49, comparable with the variation in BrU incorporation we observed in this cell type (CV = 0.46). To assess whether the differences in Br-RNA between cells correspond to variation in transcription rate, we performed FLIP analysis in a group of C23 cells, followed by BrU incorporation. This experiment showed a strong relationship between DNA-associated RNA pol II *t*_1/2_ and Br-RNA production (Figure 1F). The faster the RNA pol II was dissociating from the DNA, the more Br-RNA was produced, supporting the suggestion that variability in the rate of DNA dissociation was coupled to variability in the rate of transcript elongation.

Transcription through intact chromatin involves the removal of histone H2B in order to destabilise the nucleosome [24], and consequently the dynamic properties of histone H2B reflect transcription elongation rate [25]. We therefore analysed the rate of exchange of fluorescently tagged histone H2B as a complementary approach to assess RNA pol II elongation rates in individual cells. Half of the nuclei of Hela cells expressing histone H2B–GFP were photobleached, and the decay of the signal in the unbleached halves was analysed. H2B–GFP showed a bi-exponential decay with a short *t*_1/2_ population that exchanges in a transcription-dependent manner (Figure S4) (∼7% of the histone H2B–GFP). The *t*_1/2_ of the fast-turnover histone H2B–GFP showed a wide range of values, consistent with different cells transcribing at different speeds (Figure 1G and 1H). This was again corroborated by experiments where cells were incubated with BrU after photobleaching, showing a good relationship between H2B (*t*_1/2_) and Br-RNA production (Figure 1I). As in the case of RNA pol II, the more dynamic the exchange of H2B, the more Br-RNA was produced, and vice versa. Taken together, these results provide good evidence that transcription elongation varies significantly between different individual cells within an otherwise homogenous population.

Next, we asked whether all the elongating RNA pol II molecules in a given cell were transcribing at a similar speed. In order to analyse only the nascent transcripts we limited the BrU pulse to 15 min and immediately “froze” cells with sarkosyl [18]. We measured the intensity of multiple individual Br-RNA foci within each nucleus (Figure 1J and 1K). We plotted the CV of the intensity of these nascent transcripts (Br-RNA foci) versus the mean intensity of these foci in the same cell, and carried out the analysis in cells exhibiting different amounts of transcription (Figure 1L). The data show scant change in the CV, consistent with all the polymerases that share the same nucleus transcribing at similar speed. There, thus, appears to be a global factor coupling the transcription rates of all foci across the nucleus (the variability in the rate of expression between different foci in the same nucleus is independent of the average rate of expression in the nucleus).

To summarise, we found a marked variability in the levels of steady state incorporation of BrU in genetically identical populations (Figures 1A and S1A) (and this appears to be independent of cell cycle position; Figure S1E). We then investigated the connection with transcription rate. The RNA pol II experiments suggested that there was marked cell-to-cell variability in the rate of dissociation of RNA pol II from the DNA (even though run-on data suggested that the amount of associated RNA pol II is relatively constant between cells; Figure S2A and S2B). The H2B–GFP experiments (Figure 1H) suggested this was related to cell-to-cell variation in transcription elongation rate. Both of the bleaching experiments suggested a correlation between DNA dissociation rates, RNA elongation rates, and the levels of BrU incorporation (Figure 1). This leaves the factor responsible for this cell-to-cell variation in global transcription rate unexplored, but, as the experiments in Figure 1L show, the factor appears to be affecting all transcription foci equally in the nucleus.

### In Vitro Studies Show a Sensitive Dependence of Transcription Rate on [ATP]

**Figure 2:**
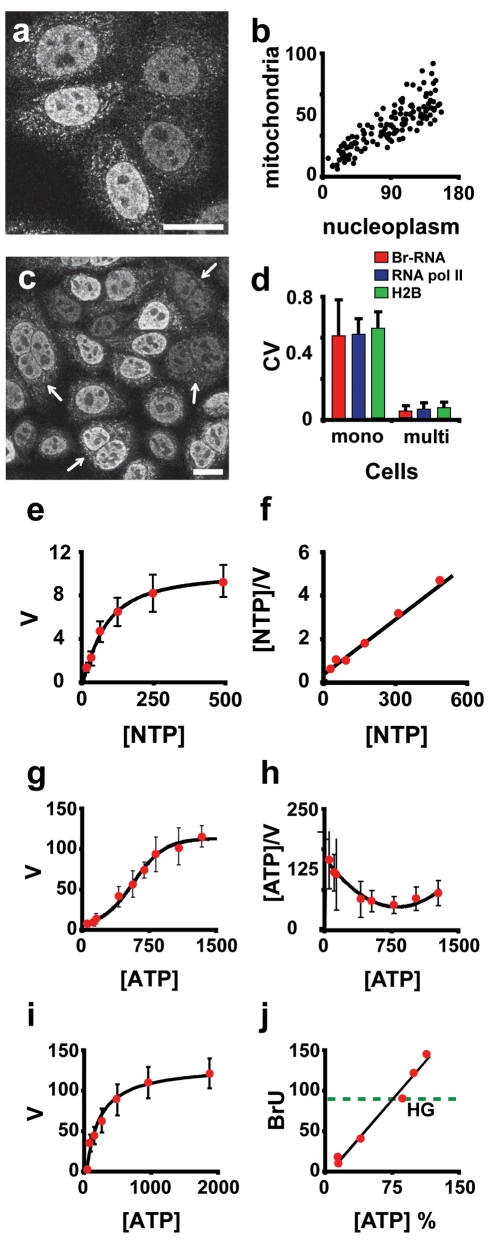
Kinetics of RNA pol II in situ: effect of NTP concentration. (A) Transcription in the nucleus and mitochondria are related. Br-RNA was immunodetected in cells after BrU incorporation. Cells with a very actively transcribing nucleus also have high levels of transcription in the mitochondria. (B) Analysis of Br-RNA signals in nucleoplasm versus mitochondria (arbitrary units) in images like that in (A) (confocal images). (C) Cells were fused using polyethylene glycol and after 2.5 h were exposed for 30 min to 2.5 mM BrU. Arrows point to fused cells, where signals in nuclei sharing the same cytoplasm present very similar intensities. (D) Analysis of the CV between nuclei of neighbouring cells (mono) and nuclei sharing the same cytoplasm (multi); CV of Br-RNA incorporation (red columns), CV of RNA pol II elongating (blue columns), and CV of the dynamics of exchange of histone H2B (green columns). (E) Study of the enzymatic kinetic properties of RNA pol II with respect to [NTP]. The assay was performed as described in Figure S5, using as a tracer the incorporation of BrUTP into the nascent transcripts of Hela cells. This panel shows the dependence of transcription rate, *V* (arbitrary units/minute) on NTP concentration (micromoles). The experimental data fit a hyperbolic curve consistent with Michaelis-Menten kinetics. (F) Plot of [NTP]/*V* versus [NTP]; the straight line obtained suggests that RNA pol II has Michaelis-Menten-like kinetics [28]. (G) Dependence of *V* on ATP concentration (micromoles) using normotonic conditions. This curve shows a sigmoidal pattern. (H) Plot of [ATP]/*V* versus [ATP] suggests that RNA pol II is allosteric with respect to ATP. The sigmoidal curve of *V* versus [ATP] suggests that the rate of BrUTP incorporation has a quadratic dependence on [ATP]. (I) Repetition of (G) but using swollen cells (hypotonic shock) in order to decondense the chromatin. Under these conditions, the kinetics of RNA pol II with respect to [ATP] appears hyperbolic. (J) Effect of intracellular [ATP] on BrU incorporation. The intracellular [ATP] was perturbed by incubation with DG (25 mM) and no glucose for 12 h, which decreases [ATP], or incubation with succinate (5 and 10 mM) for 30 min, which increases [ATP]. Green dotted line represents BrU incorporation under ATP control conditions (high glucose, 4.5 g/l). Bars = 10 µm.

Next, we investigated whether the global factor responsible for the variation in transcription rate was soluble. In a first approach we incubated cells with BrU for 30 min and analysed the intensity in the nucleus and mitochondria in individual cells. This experiment showed a strong correlation between BrU incorporation in these two compartments (Figure 2A and 2B), suggesting the factor is not nucleus specific. In a second experiment, we fused Hela cells with polyethylene glycol and after 2.5 h we carried out BrU incorporation for 30 min. This experiment showed that nuclei sharing the same cytoplasm have almost identical levels of BrU incorporation per unit of nuclear volume, showing an average CV of 0.04 (in contrast, the average CV for randomly selected pairs of nuclei from different cells was 0.50). The same was observed when the dynamic properties of RNA pol II–GFP or histone H2B–GFP were analysed in fused cells (Figure 2C and 2D) (note that in Figures 2D and S12D the CV is the average of the CVs calculated for pairs of nuclei). Both sets of experiments suggested the existence of a small soluble factor responsible for the variation. An obvious candidate is differences in substrate content (nucleotides) available to RNA pol II in each cell. This was supported by the observation that “in vitro” transcription using a fixed concentration of bromouridine triphosphate (BrUTP) as a tracer showed a much lower degree of variability than BrU incorporation “in vivo” (CV<0.10) (Figure S5). BrUTP incorporation “in vitro” was performed in permeabilized cells, guaranteeing an even concentration of precursors to all cells.

Based on these results, we sought to analyse the relationship between nucleotide precursors and BrU incorporation. However, even though we have a good knowledge of NTP concentrations in cell populations [26], there are no methods available to measure the nucleotide content in individual cells that are appropriate in this case. Instead, we studied the behaviour of RNA pol II with respect to [NTPs]. We used a nonradioactive method to measure the kinetic properties of RNA pol II, attached to the appropriate template, in the natural environment of the cell nucleus. Our method is based on measurements of the amount of incorporated BrUTP in nascent RNA, detected by immunofluorescence. We checked the dependence of the speed of transcription on different substrate concentrations. Cells were incubated with a cocktail containing different concentrations of all the NTPs except for ATP, which was fixed at a cell physiological level of 1 mM (henceforth NTP will refer to UTP, CTP, and GTP only). Plotting transcription rate, *V*, versus [NTP] yields a hyperbolic curve (Figure 2E), consistent with Michaelis-Menten kinetics with a *K*_m_ of 80±10 µM (*R* ^2^ = 0.996) (Figure 2E and 2F). This suggests that RNA pol II activity depends on the nucleotide content of the cell. However, the concentration of NTPs inside the cell is believed to be in the millimolar range [26]. From Figure 2E, this means that RNA pol II is effectively working at full speed with respect to NTPs (even if NTP concentration falls from 1 mM to 250 µM). Therefore, [NTP] is unlikely to be the factor responsible for the observed variation.

Some models for transcription in the literature have explicit and implicit energy dependences (see [27] for an example). Given this energy dependence, we also studied the RNA pol II activity with respect to [ATP] (this time fixing NTP concentration at 100 µM). The plot of *V* versus [ATP] showed a sigmoidal curve (Figure 2G). A plot of [ATP]/*V* versus [ATP] (Figure 2H) [28] emphasises this. It is thus possible that RNA pol II behaves as an allosteric enzyme (Hill coefficient of 1.5±0.34; *R* ^2^ = 0.99; *K*_m_ 870±450 µM) with respect to ATP.

An allosteric behaviour of RNA pol II has not to our knowledge been previously reported, possibly because all other studies have been performed either in vitro with purified enzymes or without the near-physiological conditions necessary to minimise the perturbation of essential macromolecular complexes. Our transcription system uses physiological salt concentrations and macromolecular crowding agents that keep the molecular complexes as close as possible to “in vivo” conditions.

The apparent allosteric behaviour of RNA pol II is consistent with evidence that active RNA pol II forms structures containing several molecules [18],[20],[29]. There are also reports of more simple viral RNA polymerase molecules that oligomerize and show cooperativity [30]. Another explanation for this allosteric behaviour could be an effect of ATP on other proteins that influence the catalytic activity of RNA pol II. Obvious candidates are remodelling factors and/or DNA helicases that are generating template for RNA pol II in an ATP-dependent manner. In this category we can find the ATPase CHD1 (chromo-ATPase/helicase–DNA-binding domain), which remodels nucleosomes in vitro and appears to function in both elongation and termination [31]. Another example is the remodelling complex SWI/SNF, which is also ATP dependent and associates with the RNA pol II holoenzyme [32]. Therefore, the activity of all these factors should affect the apparent activity of RNA pol II. To study if this was the case we decided to uncouple transcription from remodelling. We reasoned that by decondensing chromatin, remodelling factors would not limit the availability of DNA, and therefore these factors would contribute very little, if at all, to the kinetics of RNA production. We explored such a possibility by repeating the study of the relation between RNA pol II kinetics and [ATP] in swollen cells. Incubation of cells in hypotonic buffer for 10 min induced chromatin decondensation (Figure S6), and in these swollen nuclei the kinetic behaviour of RNA pol II with respect to [ATP] was hyperbolic (Figure 2I), in contrast to the sigmoidal kinetics observed in unswollen native cells. This hyperbolic behaviour with respect to [ATP] has also been reported for remodelling factor(s) [33]; the sigmoidal kinetics of RNA pol II with respect to [ATP] may be the result of two consecutive sub-processes (elongation and remodelling) with hyperbolic kinetics.

Chromatin remodelling effects have been suggested as a cause of intrinsic noise [2], so it is interesting to note their possible role in global variability. Whatever its origin, sigmoidicity seems to be dependent on the native status of these molecules on the natural template, which means that it probably reflects an in vivo scenario. As the intracellular [ATP] is believed to be ∼1 mM [26] (close to the RNA pol II *K*_m_ of ∼870 µM, found in our conditions), small fluctuations in [ATP] are likely to affect transcription elongation in vivo. (This paper is concerned with the connection between transcription rate and mitochondrial function, but we also investigated the connection between mitochondrial mass, ATP, and protein synthesis; more details can be found in Figure S13.)

We presented evidence that the global factor modulating transcription rate does so for both nuclear and mitochondrial genes (and so is not nuclear specific; Figure 2B). Fusion studies suggested this factor is small and rapidly diffusing (Figure 2D). In vitro studies indicate a sensitive dependence of transcription rate on [ATP] (at around cellular concentrations), while this is not the case for other NTPs (Figure 2E and 2G). Decondensing the chromatin eliminates this sensitivity (Figure 2H).

### Mitochondrial Mass, Membrane Potential, and [ATP] Are Linked to Transcription Rate

**Figure 3:**
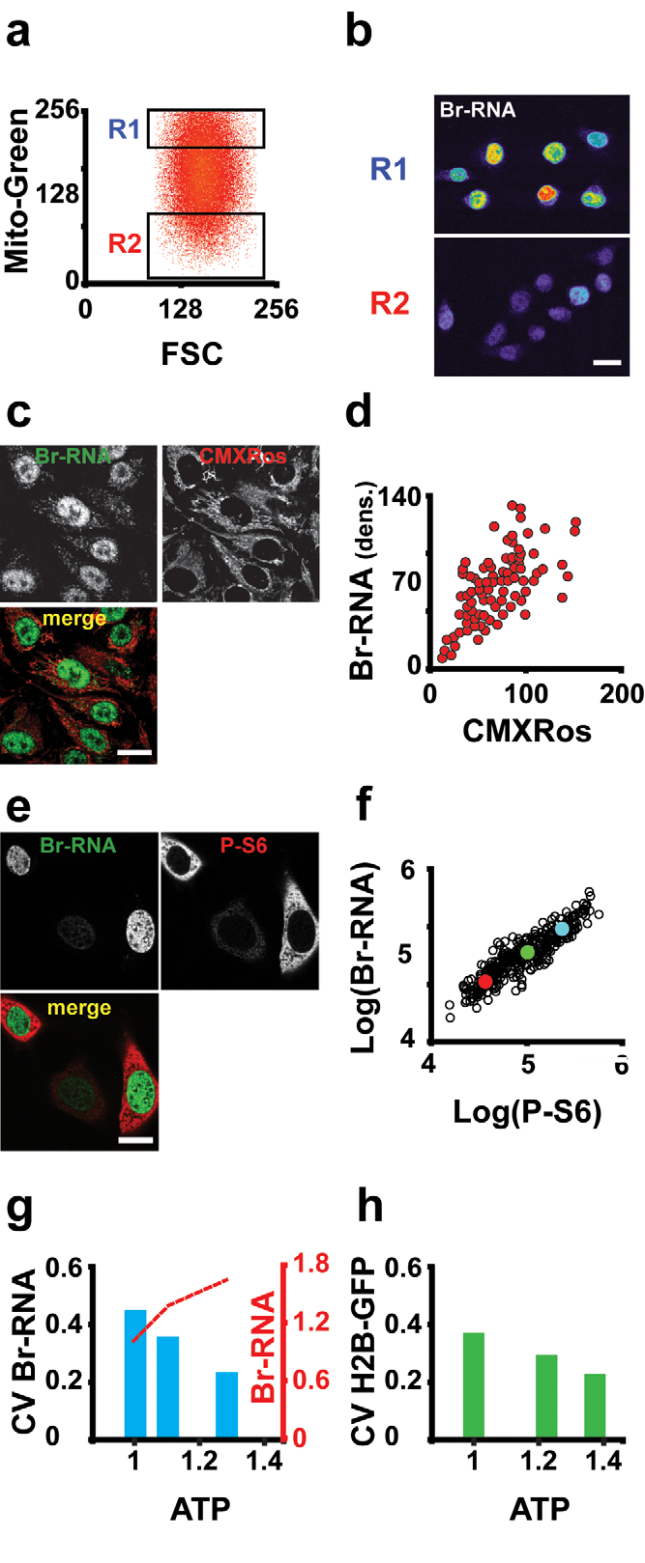
Mitochondria determine transcription elongation speed. (A) Hela cells were sorted according to the mitochondrial content after staining with MitoTracker Green FM. Two populations of cells were sorted, with a difference in mitochondrial content of around 5-fold (R1 and R2). (B) Four hours after plating, cells were incubated with BrU. These experiments show a direct relationship between mitochondrial content and both RNA production and mRNA content (quantification in S8A and S8B). (C) Cells stained with the redox-sensitive mitochodrial probe CMXRos (middle panel) and Br-RNA (left panel). (D) Quantitative analysis of images like that in (C) (arbitrary units). There is a direct relationship between CMXRos and BrU incorporation signals (confocal images). (E) Co-staining of Br-RNA and P-S6 in Hela cells. (F) Nuclear Br-RNA production under conditions of ATP depletion. Cells were incubated with DG for 12 h, and at the end of incubation 5 mM BrU was added. In this plot we superimposed the data points of three different conditions used: glucose (average: blue dot), 25 mM DG (average: green dot), or 50 mM DG (average: red dot). (G) ATP affects CV in BrU incorporation. Intracellular ATP concentration was raised by succinate incubation (5 and 10 mM) for 30 min. The CV of Br-RNA is reduced in a manner proportional to ATP (blue bars), and BrU incorporation increases in parallel to intracellular ATP increase (red dotted line). (H) CV of the speed of exchange of histone H2B–GFP. This reporter behaves very similarly to BrU, decreasing as intracellular ATP increases. Bars = 10 µm.

The experiments described above suggest that the differences seen in BrU incorporation could be a reflection of cellular heterogeneity in ATP content. Indeed, in population studies where we perturbed intracellular [ATP], we observed a direct relationship between BrU incorporation and [ATP] (Figure 2J). A similar effect was observed in the rate of dissociation (*t*_1/2_) of RNA pol II (Figure S7). By sorting cells according to their mitochondrial content (using MitoTracker Green FM dye), we found that cells with a higher transcription rate per unit volume of nuclear material also have more mitochondrial mass (Figures 3A, 3B, and S8). Using similar sorting experiments, we found evidence that a crude measure of cellular [ATP] covaried with mitochondrial content (Figure S9A and S9B). We explored this correlation further using another indicator of [ATP]. ATP is a product of mitochondrial function so we assessed the mitochondrial membrane potential (Δψ), which is the driving force for ATP production [34]. Cells were sorted according to tetramethyl rhodamine methyl ester (TMRM) levels (an indicator of Δψ), and we found evidence for a correlation with an approximate measure of cellular [ATP] (Figure S9C). Single-cell studies showed that both total membrane potential and also transcription rate are slowly varying (Figure S10; Video S1). For further discussion, see Text S1.

To study the relationship between Δψ and the rate of BrU incorporation per unit of nuclear volume we used MitoTracker Red (CMXRos), a fixable probe (TMRM is not fixable) sequestered inside the mitochondria that depends on Δψ. After incubation with both reagents we quantified the signals in individual cells. The two parameters showed a strong relationship, suggesting that total membrane potential relates to the BrU incorporation (Figure 3C and 3D). To give further support we used phosphorylated ribosomal protein S6 (P-S6) as a reporter of the energy state of the cell. P-S6 is located downstream in the mTOR pathway. mTOR is a homeostatic [ATP] sensor, and phosphorylation of its targets is dependent on ATP concentration [35]. One target of mTOR is the ribosomal S6 kinase (S6K1) that phosphorylates the ribosomal protein S6 [36]. The use of P-S6 as a reporter for energy status was validated by induction of energy stress after deprivation of glucose and incubation with deoxyglucose (DG) for 12 h, which resulted in depletion of cellular ATP ([36] and this study, data not shown). As predicted, P-S6 decreased in response to energy depletion (), working as a surrogate reporter of the energy status of the cell. Next, we incubated cells for 30 min with BrU, and after immunolabelling with BrU and P-S6 antibodies, we observed a correlation between both signals (Figure 3E), and both decreased in a manner proportional to the concentration of DG (Figure 3F). We also increased the intracellular concentration of ATP by incubation with succinate at 5 and 10 mM, which increased [ATP] to 135% of normal levels, resulting in an increase in transcription rate and reduction in transcription rate variability (assessed by measuring total nuclear BrU incorporation and H2B *t*_1/2_ exchange (Figure 3G and 3H).

If mitochondrial activity is coupled to variability in transcription rate, then changing mitochondrial function by altering the presence of anti- or prooxidants might affect this rate and its variability. We undertook studies using the antioxidants dithiothreitol (DTT) and MnTMPyP and prooxidants diamide and N-ethylmaleimide (NEM) (Figure S12). These studies suggested that the presence of antioxidants increases transcription rate and reduces rate variability, with the opposite holding for prooxidants (Figure S12B and S12C). For further discussion, see Text S2.

In summary, we find evidence suggesting that transcription rate per unit volume of nuclear material covaries with the mitochondrial mass of cells (Figures 3A, 3B, and S8). We also found that a measure of membrane potential (integrated over the cell) correlated with transcription rate per unit volume (Figure 3D). By modulating intercellular nutrients we modulated intracellular [ATP] and found that this also correlated with the degree of BrU incorporation (Figure 2J). Further indirect studies gave support to this connection between ATP levels, mitochondrial mass, and transcription rate (Figures 3F–3H, S9, and S12).

### Mitochondrial Segregation Noise Modulates Cell Cycle Length and Contributes to Cell-to-Cell Variability in Mitochondrial Mass

**Figure 4:**
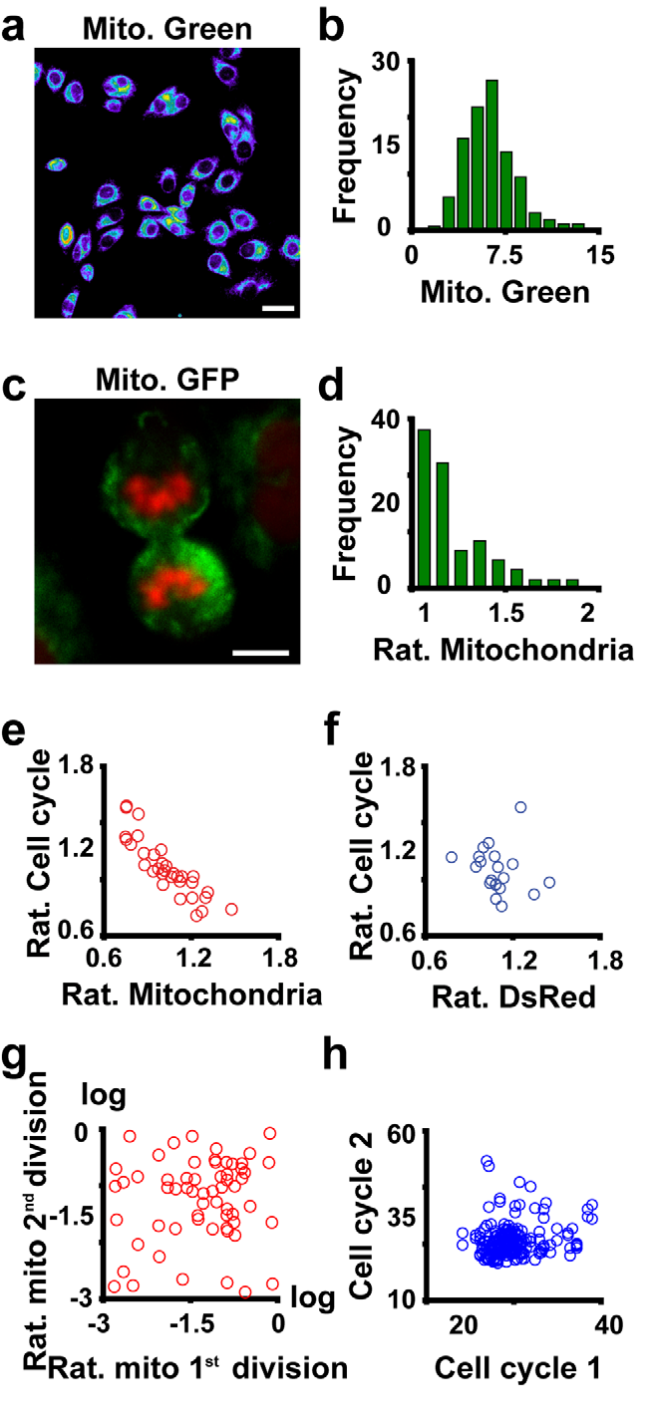
Asymmetric segregation of mitochondria. (A) Visualisation of mitochondria with MitoTracker Green FM. (B) Quantitative analysis of the mitochondrial content in samples stained as in (A) (*n* = 800). (C) Asymmetric segregation of mitochondria in mitotic cells. Cells in telophase show asymmetric segregation of mitochondria (green) and DNA (red). (D) Quantitative analysis of the ratio (rat.) of mitochondrial content between daughter cells (*n* = 300). (E) Mitochondrial content is correlated with the cell cycle length, as demonstrated by the plot of the ratio of cell cycle length versus the ratio of mitochondrial content between daughter cells at division (*R* ^2^ = 0.8). (F) A weak correlation was found between the ratio of daughter cell volumes at birth (measured by the soluble protein DsRed) and their relative cell cycle lengths (*R* ^2^ = 0.06). (G) Analysis of the difference in mitochondrial content between sister cells after the first division (ratio of intensities of mito-YFP) and the second division. No relationship could be observed between segregation of mitochondria in two consecutive mitotic events. (H) Analysis of the interdivision time between consecutive cell cycles in individual cells. Bars: (A), 10 µm; (C), 5 µm.

In order to understand the origin of cell-to-cell differences in transcription rate, and given the observed connection between transcription rate and mitochondrial mass, we measured the mitochondrial content in Hela cells using MitoTracker Green FM dye. This staining demonstrates that Hela cells are heterogeneous in terms of mitochondrial content (Figure 4A and 4B). We investigated the asymmetric segregation of mitochondria between daughter cells as a possible source of this heterogeneity. We used for this analysis a stable cell line containing mitochondria tagged with yellow fluorescent protein (YFP). A plasmid encoding subunit VIII of cytochrome c oxidase fused with YFP was transfected into an epithelial-like human cell line derived from a bladder carcinoma (ECV304). The tagged subunit is incorporated into mitochondria, diffusing rapidly throughout the interior of the mitochondrion [37]. This makes this chimeric protein an ideal reporter to study the behaviour of mitochondrial mass at mitosis. We focused on cells in telophase or late mitosis (Figure 4C), where we measured the mitochondrial mass for each daughter cell as the integrated intensity of the mitochondrial signal [38]. This analysis showed that cells generically segregate mitochondria in an uneven manner (Figure 4C and 4D).

Given the observation that mitochondrial mass segregates asymmetrically, one can ask whether this is relevant to cell physiology. Cell tracking experiments showed that mitochondrial content at mitosis correlates with cell cycle length. The daughter cells with more mitochondria progressed through the cell cycle proportionately faster than their sisters (Figure 4E). To rule out the trivial explanation that asymmetry in mitochondrial content was an effect of asymmetry in the volume of daughter cells, we used ECV304 cells expressing DsRed, which is a soluble protein and distributes evenly throughout the cell. The analysis of the ratio of DsRed between daughters (ratio of cell volumes) versus the ratio of time to complete a cell cycle did not show a clear relationship (Figure 4F), making it unlikely that asymmetries in the volume of daughter cells are the principle cause. We also found that daughters that inherit more mass than their sisters also have a higher rate of translation of some proteins (Figure S13A); this further suggests that the uneven inheritance of mitochondria has an effect through the cell cycle. For further discussion, see Text S3.

Since mitochondrial segregation is correlated with variation in cell cycle length, one might want to understand how mitochondrial partition at birth is controlled. We analysed whether the process of mitochondrial segregation had a memory. We measured the mitochondrial content ratio between daughters at birth (F1), followed each daughter for one cell cycle, and measured the mitochondrial content ratio of daughters in generation F2. When we compared F1 versus F2 in terms of asymmetry, there was no clear relationship (Figure 4G). The time to division of generation F1 cells was largely independent of the interdivision times of respective F2 cells (Figure 4H). This gives us a more refined view of the stochastic character of mitochondrial segregation.

We have found evidence for variability in mitochondrial mass within a population (Figure 4A). A possible cause is asymmetric segregation of mitochondrial mass at division (Figure 4C and 4D). Daughter cells that inherit more mitochondrial mass progress through their cell cycles faster and can show a faster rate of protein synthesis (Figures 4 and S13A–S13D). We found no strong evidence for a dependence between one cell cycle duration and the next (Figure 4G and 4H).

### Summary

This paper investigated the connection between two forms of cellular variability: variation in mitochondrial mass and variation in global transcription rate. We found marked heterogeneity in the amount of mitochondrial mass present in cells (Figure 4B) and evidence for an origin of this variability in the stochastic partition of mass at point of division (Figure 4D). We further found that this variation has a cell physiological correlate: daughters that inherit relatively smaller amounts of mitochondrial mass than their sisters have longer cell cycles (Figure 4E). We also presented evidence for global (Figure 1L) variability in transcription rate (Figures 1E, 1H, and S1A). While our experiments suggest that the numbers of bound RNA polymerases are constant and transcription rate variability is independent of cell cycle stage (Figures S1E, S2, and S5), they also suggest that a small diffusing factor may be responsible for this global transcription rate modulation (Figure 1D).

Given the above, we hypothesized, first, that there was aconnection between global transcription rate variability and variability in cellular mitochondrial content and, second, that this was mediated by variation in the fast-diffusing factor ATP. Studies in permeabilized cells (Figure 2G) found a sensitive dependence of transcription rate on [ATP]. In vivo perturbation studies also found a correlation between cellular [ATP] and transcription rate (Figure 2J). We found that cells with more mitochondrial mass transcribe faster per unit volume of nuclear material (Figure 3B). We further found that those with higher total membrane potential (as indicated by CMXRos) transcribed faster (Figure 3D). We also found evidence correlating levels of ATP with mitochondrial mass and total membrane potential (Figure S9B and S9C). Finally, we found that perturbing mitochondrial function with anti- or prooxidants perturbed transcription rate variability (Figure S12).

Studies thus far have left our understanding of the origins of global variability in gene expression in higher eukaryotes unclear [2]. This paper suggests that cell-to-cell variability in mitochondria is coupled to cell-to-cell variability in global transcription rate.

## Materials and Methods

### Transcription and Immunofluorescence

For in vivo transcription, cells were incubated in the presence of different concentrations of BrU (Sigma) for different times (stated in figure legends). Incubation for 1 h with 100 µM DRB or for 1 h with 1 µg/ml actinomycin D prior to BrU incubation abolished BrU incorporation completely (data not shown).

For individual transcript analysis, Hela cells were grown on coverslips at low density then incubated for 15 min with 5 mM BrU, washed with PBS, and treated with 0.375% sarkosyl, 25 U/ml ribonuclease inhibitor, 10 mM EDTA, and 100 mM Tris-HCl (pH 7.4) for 10 min at 20°C. Next, coverslips were tilted to allow the cell content to run out for 5 min. Samples were air-dried and fixed with 4% paraformaldehyde for 10 min and processed for Br-RNA detection.

For transcription in vitro we used the conditions described in [18] plus 5% Ficoll 400.

For detection of primary transcripts, we used mouse anti-IdU/BrdU (5 mg/ml; Caltag Laboratories). Secondary antibodies were donkey anti-mouse IgG tagged with Cy3 (1/200 dilution; Jackson ImmunoResearch). The immunodetection procedure was performed as described in [18],[19]. DNA was stained with 200 nM TO-PRO-3 (Molecular Probes) for 5 min, then slides were mounted in Vectashield (Vector Laboratories), and images were collected using a Radiance 2000 confocal microscope (BioRad Laboratories). Intensities in the nucleoplasm were measured using EasiVision software (Soft Imaging Systems) and data exported to Excel (Microsoft) for analysis.

For cell fusion experiments, Hela cells were grown on coverslips to 80% confluence. Cells were fused using polyethylene glycol as described by Schmidt-Zachmann et al. [39]. After 2.5 h cells were incubated with 2.5 mM BrU for 30 min and then immunolabelled as described above.

### FLIP

A clone stably expressing GFP–RNA pol II (C23) [22] was cultured at 39°C, and images were collected with the microscope stage heated to 39°C. Fluorescence images were collected using a confocal microscope (Zeiss LSM 510 META), with an EC PlnN 40×/1.3 oil objective, with the pinhole completely open. We selected a rectangle at the bottom half of each nuclei where we applied 100% laser power, in order to bleach all the fluorescent molecules in the rectangle. This operation was repeated every 5 s for a period of 1,200 s, and we analysed the decay of the fluorescence in the unbleached top half. Fluorescence intensity was analysed in MetaMorph 6.1 (Universal Imaging). Curves were analysed using Sigma Plot 8.0 for Windows. For the analysis we assumed that there were two populations, freely diffusible, bound to DNA and fully engaged in transcription. For the fitting we allowed the two components to optimise with no restriction. Data were fitted to two populations with exponential decay (always *R* ^2^>0.99). Fixing the slow population to an average speed rendered unacceptable fittings with the second population. We were concerned with the possible artefacts induced by FLIP. Therefore, transcription “run on” experiments were performed on photobleached cells, which demonstrated no alteration in the transcription pattern or intensity in the bleached area (data not shown).

Hela cells expressing histone H2B–GFP [25] were used to study the dynamics of histone H2B. FLIP was performed as for C23 cells, but the time was reduced to 10 min of photobleaching and the temperature was set at 37°C. The decay curves can be fitted to a bi-exponential decay. The two initial points were discarded because they correspond with the free population of histone H2B. One possible problem with the use of the exchange of histone H2B–GFP as a transcription reporter is the impact of its overexpression. However, in the cell line used, histone H2B–GFP represents 10% of all cellular histone H2B [25]. The production of natural histone H2B is reduced in preserving the normal amount of histones, which means that no overexpression occurs in this cell line [25]. The fraction of free histone in the cell line used is around 1% of the total H2B–GFP, which corresponds with the fraction bleached in the first two cycles of bleaching. This population was not considered for the analysis. Even if any hyperexpression occurs in this cells, only the fraction of molecules bound to DNA and not the *t*_1/2_ will be affected, which is the parameter studied. In agreement with this interpretation we did not observed a correlation between the initial fluorescence before bleaching of histone H2B–GFP and *t*_1/2_ (Figure S4C).

### Cell Sorting

Tripsinized Hela cells were stained with MitoTracker Green FM dye (Molecular Probes) for 15 min in DMEM or TMRM (Molecular Probes) for 30 min, following manufacturer guidelines. Then, cells were sorted on a fluorescence-activated cell sorter (MoFlo; DakoCytomation) to purify populations of cells with different mitochondrial content or membrane potential.

### MitoTracker, MitoSox Staining, ATP Depletion, and Antioxidant Treatments

MitoTracker Red (CMXRos) was used following the manufacturer guidelines (Molecular Probes). Cells were stained for 10 min in vivo after being grown in BrU for 30 min. Br-RNA was detected as previously described. For superoxide detection cells were incubated with 20 nM for 12 h with MitoSox (Molecular Probes) and then grown in BrU for 30 min. Cells were analysed using wide confocal cytometry [38].

ATP depletion experiments were carried out by incubation of cells for 12 h with different concentrations of DG (Sigma). In another set of experiments ATP was depleted by incubation with 10 mM sodium azide (Sigma) and 6 mM DG in HBSS for 1 h (BioWhittaker). ATP concentration was determined using the kit ATP Bioluminescence Assay Kit HS II (Roche) following manufacturer instructions.

For antioxidant treatments, cells were incubated for 18 h with MnTMPyP (CalBiochem) or DTT (Sigma). MnTMPyP was used at 50, 25, and 12.5 µM. DTT was used at 1,000, 500, 250, and 125 µM. GSH was depleted by incubation with 200, 100, or 50 µM diamide (Sigma) for 2 h.

### Construction of the CE–mitoRFP-W, llx–GFP, llx–Emerald, and llx–Cherry Vectors

CE–mitoRFP-W vector was generated from the pHR-SIN-CSGW vector [40] by exchanging the SFFV promoter for a human EF1a promoter and the GFP reporter for mitochondrial DsRed2 isolated from pDsRed2-Mito (Clontech). Lenti lox vector expressing GFP, Emerald, or Cherry was generated as described in [41].

### Preparation of Lentiviruses and Lentiviral Infections

Lentiviruses were pseudotyped with the vesicular stomatitis virus G (VSVG) protein by transient transfection of 293T cells [41]. Viral stocks were prepared by ultracentrifugation, and viral particles were used for Hela H2B–GFP infection; 2 wk after infection a clone was selected.

### Cell Tracking

For in vivo analysis, cells were plated in a 48-well plate at low cell density, and left for 12 h to attach. Then the plates were transferred to the Cell IQ platform (Chip-Man Technologies). Images were recorded every 30 min, for at least 6 d. Images were analysed using MetaMorph 6.1. After completion of mitosis, the ratio of the integrated intensity of the fluorescent signal between daughter cells was measured as described in [38].

## Supporting Information

Figure S1 **Transcriptional noise stabilisation.** (A) Distribution of Br-RNA levels (arbitrary units) following a 30-min pulse of BrU. (B) Time course analysis of BrU incorporation in Hela cells. The values (arbitrary units) displayed were obtained from images as shown in Figure 1A. For each time point at least 750 cells were analysed. (C) Analysis of the fluctuation of CV in Br-RNA production over the time. After 30 min of incorporation the CV becomes stable. (D) Variation in whole-nucleus BrU incorporation during the cell cycle. This panel shows the distribution of Br-RNA versus DNA content using wide confocal cytometry [38]. (E) Analysis of the CV in Br-RNA at the different cell cycle phases.
(0.10 MB TIF)

Figure S2 **Transcription in different cell types.** (A) In this experiment we carried out “run on” experiments in different cell types as described in Materials and Methods. In the first column we show the transcription signal; in the second is the DNA staining using TO-PRO-3. The intensity of the Br-RNA fluorescence in the nucleoplasm of the different cell types is almost identical. (B) Table showing the quantitative analysis of Br-RNA signal in confocal sections of the nuclei of the different cell types analysed in (A). Notice the low variability observed in the intensities as well as the almost constant value for the average intensity across the different cell types. Bar = 10 µm.
(0.59 MB TIF)

Figure S3 **FLIP Analysis of the kinetic properties of RNA pol II–GFP.** (A) This panel displays the profile of decay of 40 individual cells. Insert, average distribution of all these cells. (B) Best fit analysis of RNA pol II–GFP dynamics. Experimental decay curve from FLIP experiments (*n* = 60). (C) Best fit plots to one (green) or two (orange) exponential components. (D) Residual analysis of the best fit curves. This graph shows that the two-exponential-component curve has the best fit.
(0.65 MB TIF)

Figure S4 **Dynamic properties of histone H2B–GFP.** (A) FLIP analysis shows the presence of two populations of histone H2B–GFP (best fit analysis of two exponential decays). One population lasts for several cycles of photobleaching with a *t*_1/2_ of approximately 11 min (∼7% of the total signal) that is sensitive to DRB treatment and therefore is the population exchanged in a transcription-dependent manner. The red curve shows the decay of control cells, the blue curve shows the effect of DRB treatment. The second population is insensitive to transcription inhibitors and is exchanged slowly. (B) Log plot of fluorescence intensity of histone H2B–GFP versus time. (C) Analysis of histone H2B–GFP exchange rate versus histone H2B–GFP expression level. No correlation is evident, which suggests that expression of this reporter does not induce any artefact. This supports the use of the dynamic properties of H2B–GFP as a reporter for RNA pol II transcription elongation.
(0.15 MB TIF)

Figure S5 **“Run on” assays.** (A) Incorporation of BrUTP into the nascent transcripts of permeabilized Hela cells at three consecutive time points. After immunolabelling we observed the Br-RNA signal as small foci (left panel) distributed through the nucleoplasm (middle panel). (B) Quantitative analysis of the fluorescence intensity in the nucleoplasm of at least 200 cells like the ones shown in panel (A). The intensity of Br-RNA increases in a manner proportional to the run-on time. This graph presents data acquired with 100 µM NTP concentration. (C) Validation of the immunofluorescent approach to monitor transcription elongation. In this panel we plot the intensity of fluorescence obtained from experiments like the one shown in (B) against P^32^GTP incorporation into nascent RNA in cells [18], using the same incubation times. Bar = 10 µm.
(1.38 MB TIF)

Figure S6 **Chromatin condensation.** (A) Chromatin in intact cells with clear areas of condensed and decondensed chromatin. (B) Cell nucleus after 10 min of exposure to 0.25× PBS: we can observe a progressive decondensation of chromatin (more homogeneous grey staining) with a reduced difference between dark and bright areas.
(1.18 MB TIF)

Figure S7 **Energy and transcription elongation.** (A) Energy depletion affects RNA pol II elongation. ATP was depleted by incubation of C23 cells with 10 mM sodium azide and 6 mM DG for 30 min in HBSS. This treatment reduces ATP concentration in cells by 95% (*p*>0.99). (B) The analysis of the half-life of the elongating form of RNA pol II shows a very strong increase: RNA pol II is almost stalled at the DNA.
(0.06 MB TIF)

Figure S8 **Connecting transcription rate with mitochondrial mass.** For the sorted cells in Figure 3A we looked at (A) total BrU incorporation in the cells (4 h after plating, cells were incubated with BrU) (arbitrary units) and (B) the amount of BrU incorporation per unit volume of nuclear volume in populations R1 and R2 (arbitrary units).
(0.22 MB TIF)

Figure S9 **Mitochondria and energy content.** (A) Cells sorted according to mitochondrial content. Four regions with different mitochondrial content were sorted (boxes). Interestingly, we found that the average volume of cells in each of these fractions was approximately constant. Making the crude assumption that the average cellular volume in all four fractions is the same, ATP per average cell in each of the four regions is an approximation for [ATP] (B) ATP content was determined in these fractions. The approximate concentration of ATP per cell increases with the mitochondrial content. (C) Analysis of approximate [ATP] in cells sorted according to TMRM content. Again, a good relation is shown.
(1.11 MB TIF)

Figure S10 **Energy potential and RNA pol II elongation are slowly varying over the time.** (A) TMRM staining of Hela cells. (B) TMRM intensity fluctuation in individual cells. Images like that in (A) were taken every 20 min. (C) Hela cells expressing histone H2B–GFP were bleached for 10 min and left 50 min to recover. This bleaching cycle was repeated four times. The loss in fluorescence was measured in the unbleached part of the cell nucleus, and the faster exchanging population of histone H2B–GFP was analysed. (D) The dynamic properties of H2B–GFP do not change much over the 4 h studied, consistent with the stability in the mitochondrial potential. Bars = 10 µm.
(0.96 MB TIF)

Figure S11 **Integrated intensity of P-S6 signal in cells depleted of ATP.** ATP was depleted by 12 h of incubation of cells in medium with glucose (Gluc.) and with either 25 or 50 mM DG. P-S6 appears to be a good indicator of ATP concentration in the cell.
(0.60 MB TIF)

Figure S12 **Modulating mitochondrial function modulates transcription rate.** (A) The functional status of mitochondria affects transcription. Cells were incubated with MitoSox, a probe for superoxide anion detection, and then with BrU. (B) Variability in Br-RNA after different treatments. Cells were treated for 18 h with MnTMPyP (MnTP, 25 µM), DTT (125 µM), or 2 h with diamide (50 µM). Transcription variability is reduced with the antioxidants and increased with the prooxidant. (C) Effect of DTT on transcription in vitro with controlled [ATP]. DTT increases transcription rate (millimolar concentration); NEM (250 µM) reduces transcription drastically. (D) Analysis of BrU incorporation variability in the bulk population (column C), between sister cells (column C, sisters), between sister cells treated for 12 h with 125 µM DTT (column D, sisters), and between sisters cells expressing mito-YFP after normalisation by the mitochondrial content (column M, sisters). Cells were considered at all points in their cycles. The data presented are the average CV from three independent experiments.
(0.75 MB TIF)

Figure S13 **Protein synthesis and gene expression are dependent on ATP and mitochondrial content.** (A) Production of mito-YFP in two daughter cells with different mitochondrial content. The mito-YFP signal grows in an exponential manner (*R* ^2^>98%). A cell with more mitochondrial content produces mito-YFP faster and completes the cell cycle in a shorter time (blue circles) than a cell with fewer mitochondria (red circles). (B) Production of DsRed protein in two daughter cells with different volume (DsRed content). The signal for DsRed grows in an exponential manner (similar to mito-YFP). (C) Relationship between the ratio of rates of protein synthesis (mito-YFP) and the mitochondrial content between daughter cells. The rate of protein synthesis was calculated as the slope of the log transformation of the mito-YFP signal growth (G). This shows that the ratio of rate of protein synthesis depends on the ratios of mitochondrial content at division. (D) Relationship between the ratio of rates of protein synthesis (DsRed) and the ratio of cellular volume between daughter cells. The ratio of rates of protein synthesis is largely independent of the cellular volume ratios. (E) Visualisation of protein synthesis by bodipy-Lys-tRNA incorporation into nascent proteins in the presence of 1 mM ATP. Bodipy is incorporated mainly into the cytoplasm of Hela cells. (F) Cells incubated with the translation cocktail lacking ATP. (G) Effect of 1 mM cycloheximide on translation. (H) Quantitative analysis of nascent translation in images like the ones displayed in (E–G) (*n* = 500 cells in each group). This analysis confirms that bodipy is incorporated into proteins. (I) Analysis of the velocity of translation as a function of [ATP]. This curve shows a typical Michaelis-Menten kinetic pattern. (J) Plot of model data showing the dependence on [ATP] of the different processes involved in gene expression: translation (green), transcription (blue), and transcription and translation combined (red). This analysis shows that the process of gene expression, understood as the combination of transcription plus translation, is highly dependent on [ATP]. (K) A dual reporter study to probe protein level fluctuations. Hela cells co-expressing Emerald and Cherry show a good correlation. This panel shows the analysis of intensities of both proteins in individual cells under control conditions. (L and M) We considered the Emerald versus Cherry scatter plot under four different treatments (DTT at 0.5 and 1 mM, diamide at 100 and 200 µM, as well as control; original data displayed in Figure S14) and in each case found the best fit line (corresponding to the axis of reporter covariation or extrinsic variation). We then considered the position of data points along this axis (projecting the data points onto the best fit line). We then found the distribution of these projected positions and found the mean and interquartile range (this last being a measure of how spread out the distributions were along this axis). Error bars indicate the standard deviation of 1,000 bootstrap resamples.
(1.45 MB TIF)

Figure S14 **Dual reporter level fluctuations.** Hela cells co-expressing Emerald and Cherry, as in Figure S13K–S13M. These panels show the analysis of intensities of both proteins in individual cells standardised by the corresponding mean value. (A) 1 mM DTT, (B) 0.5 mM DTT, (C) 100 µM diamide, (D) 200 µM diamide.
(0.12 MB TIF)

Text S1 **Supplementary text for Figure S10.** Membrane potential is slowly varying over time and so is transcription rate.
(0.03 MB DOC)

Text S2 **Supplementary text for Figure S12.** Perturbing mitochondrial function perturbs transcription rate and variability.
(0.03 MB DOC)

Text S3 **Supplementary text for Figure S13.** Mitochondrial variation and translation variation.
(0.03 MB DOC)

Video S1 **Hela cells loaded with TMRM and incubated for 1 h, then transferred to fresh medium.** The images show that the signal is very stable, with no fluctuations in individual cells during the recording.
(2.44 MB AVI)

## Abbreviations

Br-RNA: RNA containing bromouridine
BrU: bromouridine
BrUTP: bromouridine triphosphate
CV: coefficient of variation
DG: deoxyglucose
DTT: dithiothreitol
FLIP: fluorescence loss in photobleaching
NEM: N-ethylmaleimide
NTP: nucleotide triphosphate
P-S6: phosphorylated ribosomal protein S6
RNA pol II: RNA polymerase II
TMRM: tetramethyl rhodamine methyl ester
YFP: yellow fluorescent protein
