# Supplementary figures and images for: Connecting Variability in Global Transcription Rate to Mitochondrial Variability
Source: PLoS Biol. 2010 Dec 14;8(12):e1000560. doi: 10.1371/journal.pbio.1000560 (PMC3001896; doi:10.1371/journal.pbio.1000560)

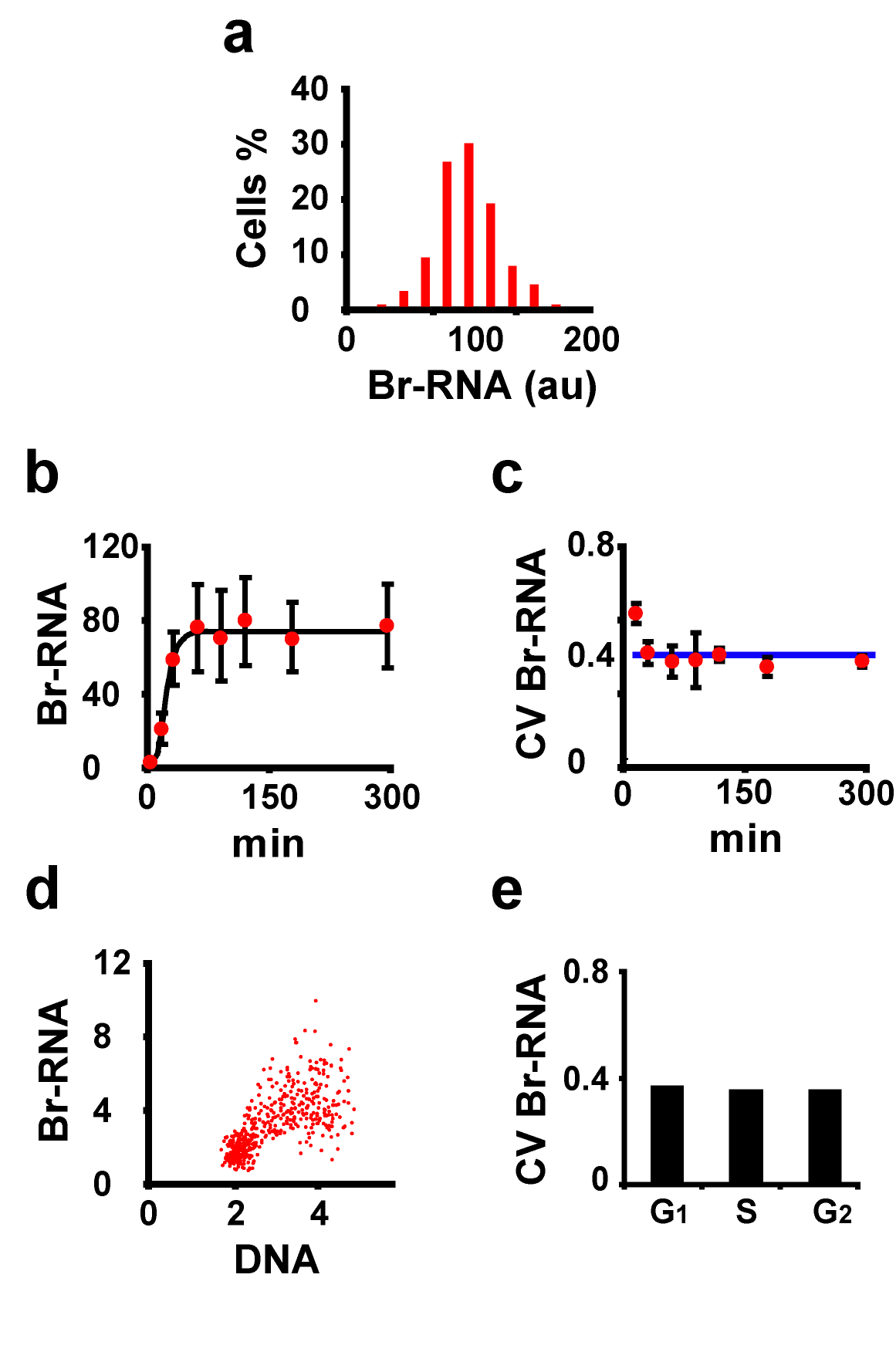

Supplement: Figure S1 — Transcriptional noise stabilisation. (A) Distribution of Br-RNA levels (arbitrary units) following a 30-min pulse of BrU. (B) Time course analysis of BrU incorporation in Hela cells. The values (arbitrary units) displayed were obtained from images as shown in Figure 1A. For each time point at least 750 cells were analysed. (C) Analysis of the fluctuation of CV in Br-RNA production over the time. After 30 min of incorporation the CV becomes stable. (D) Variation in whole-nucleus BrU incorporation during the cell cycle. This panel shows the distribution of Br-RNA versus DNA content using wide confocal cytometry [38]. (E) Analysis of the CV in Br-RNA at the different cell cycle phases. (0.10 MB TIF) [file pbio.1000560.s001.tif]

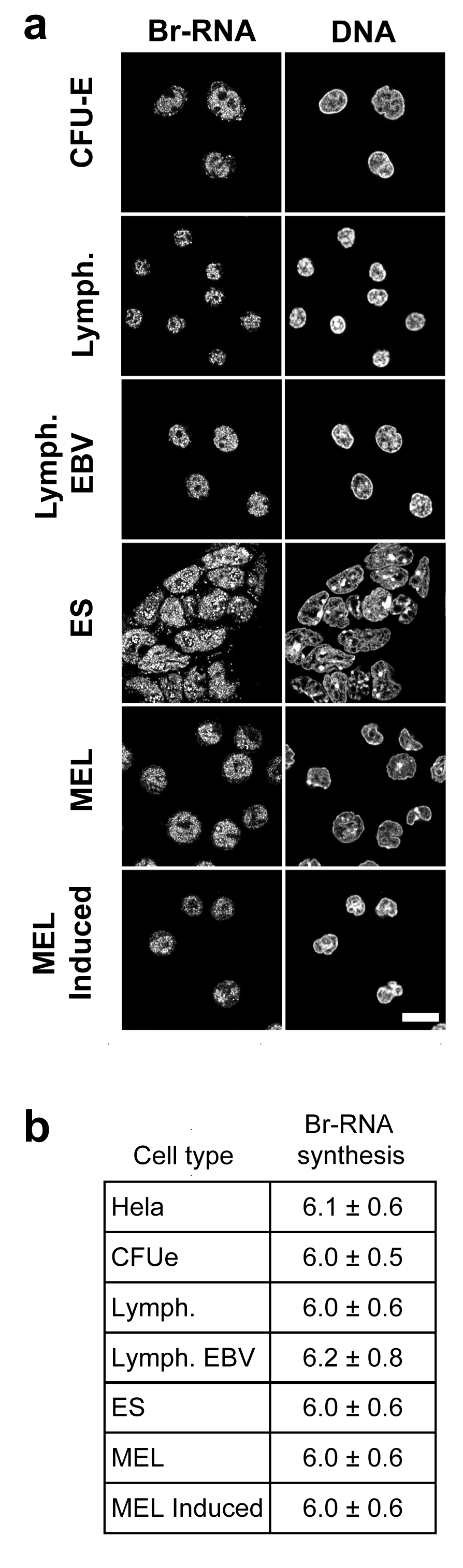

Supplement: Figure S2 — Transcription in different cell types. (A) In this experiment we carried out “run on” experiments in different cell types as described in Materials and Methods. In the first column we show the transcription signal; in the second is the DNA staining using TO-PRO-3. The intensity of the Br-RNA fluorescence in the nucleoplasm of the different cell types is almost identical. (B) Table showing the quantitative analysis of Br-RNA signal in confocal sections of the nuclei of the different cell types analysed in (A). Notice the low variability observed in the intensities as well as the almost constant value for the average intensity across the different cell types. Bar = 10 µm. (0.59 MB TIF) [file pbio.1000560.s002.tif]

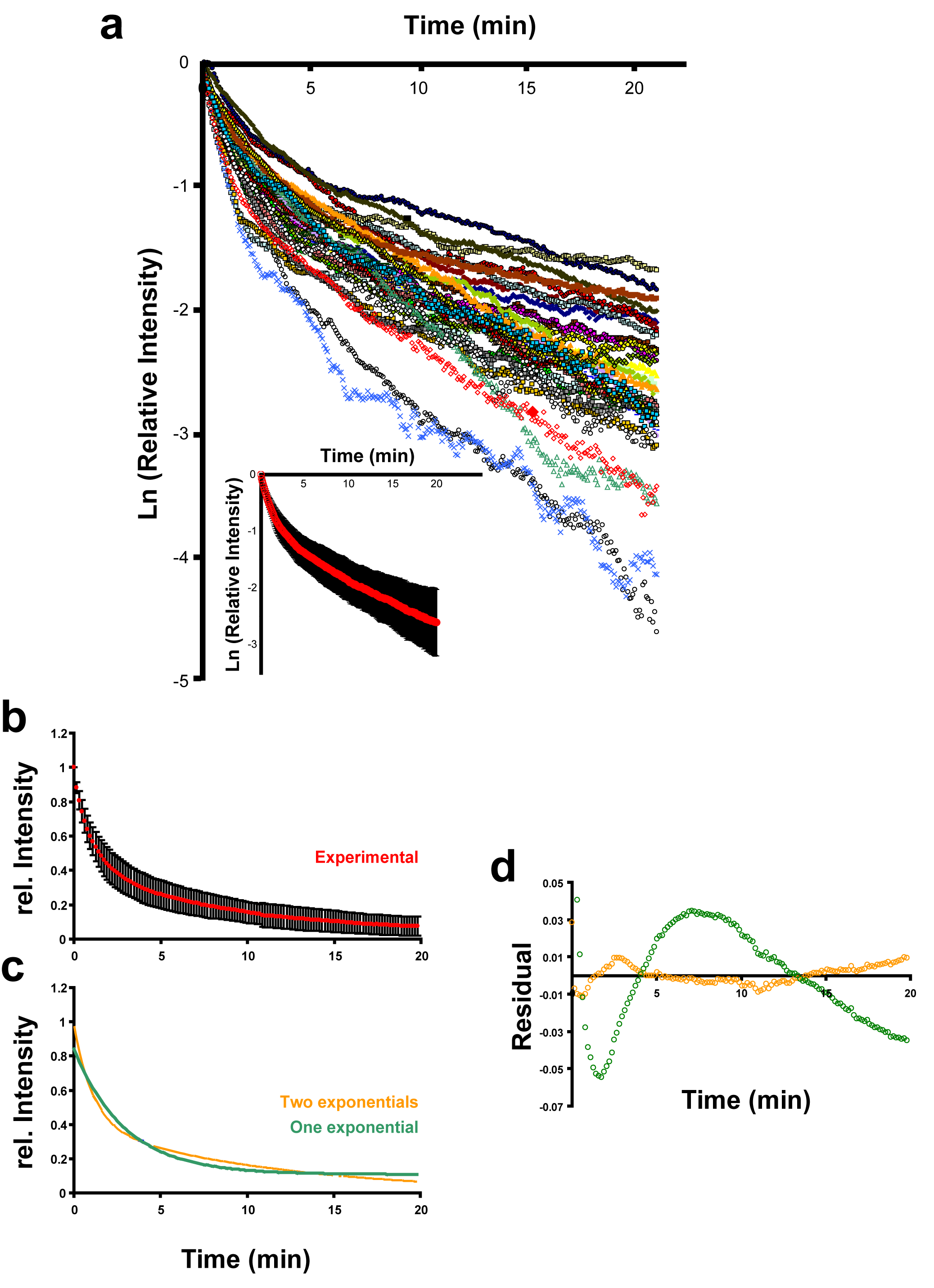

Supplement: Figure S3 — FLIP Analysis of the kinetic properties of RNA pol II–GFP. (A) This panel displays the profile of decay of 40 individual cells. Insert, average distribution of all these cells. (B) Best fit analysis of RNA pol II–GFP dynamics. Experimental decay curve from FLIP experiments (n = 60). (C) Best fit plots to one (green) or two (orange) exponential components. (D) Residual analysis of the best fit curves. This graph shows that the two-exponential-component curve has the best fit. (0.65 MB TIF) [file pbio.1000560.s003.tif]

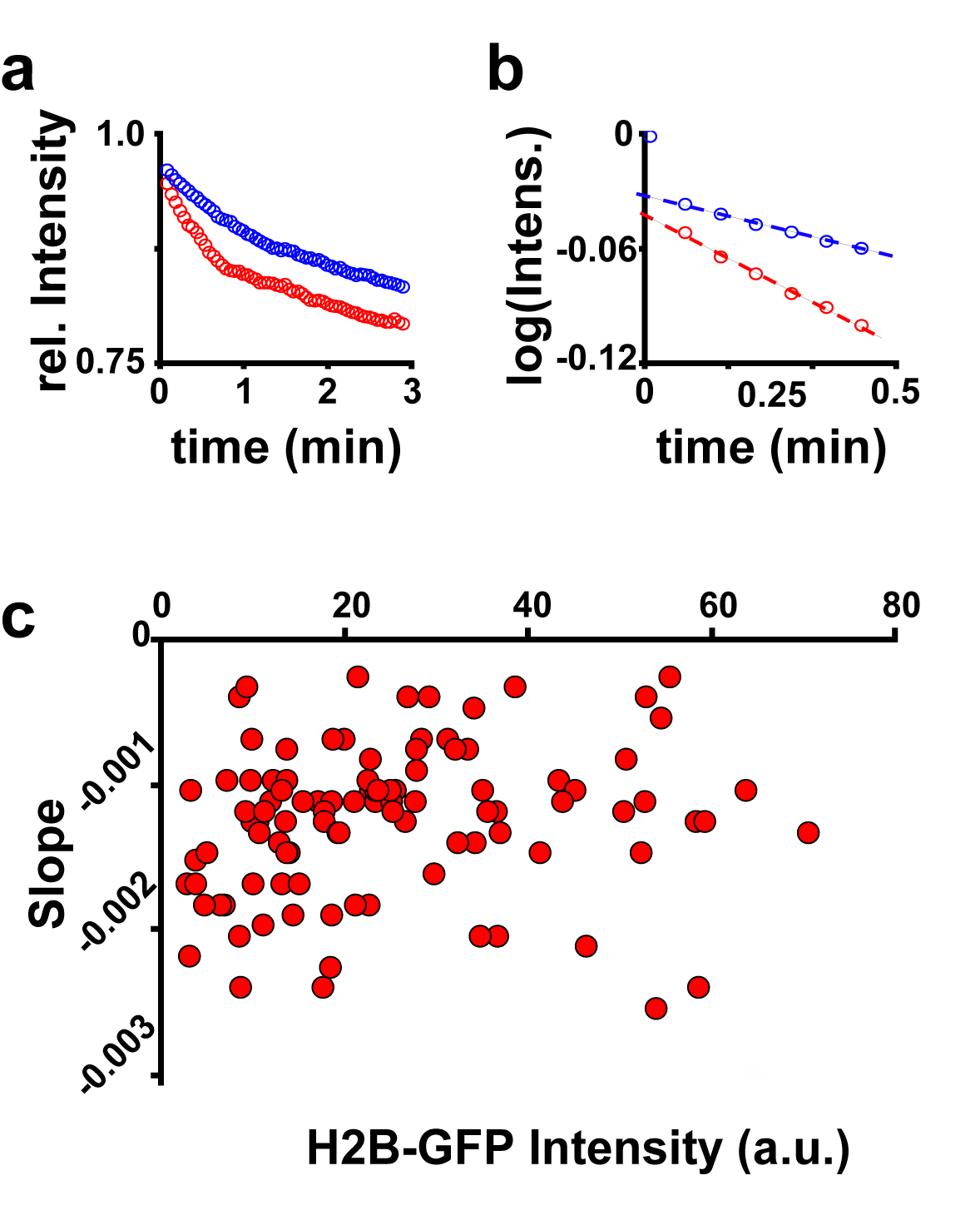

Supplement: Figure S4 — Dynamic properties of histone H2B–GFP. (A) FLIP analysis shows the presence of two populations of histone H2B–GFP (best fit analysis of two exponential decays). One population lasts for several cycles of photobleaching with a t 1/2 of approximately 11 min (∼7% of the total signal) that is sensitive to DRB treatment and therefore is the population exchanged in a transcription-dependent manner. The red curve shows the decay of control cells, the blue curve shows the effect of DRB treatment. The second population is insensitive to transcription inhibitors and is exchanged slowly. (B) Log plot of fluorescence intensity of histone H2B–GFP versus time. (C) Analysis of histone H2B–GFP exchange rate versus histone H2B–GFP expression level. No correlation is evident, which suggests that expression of this reporter does not induce any artefact. This supports the use of the dynamic properties of H2B–GFP as a reporter for RNA pol II transcription elongation. (0.15 MB TIF) [file pbio.1000560.s004.tif]

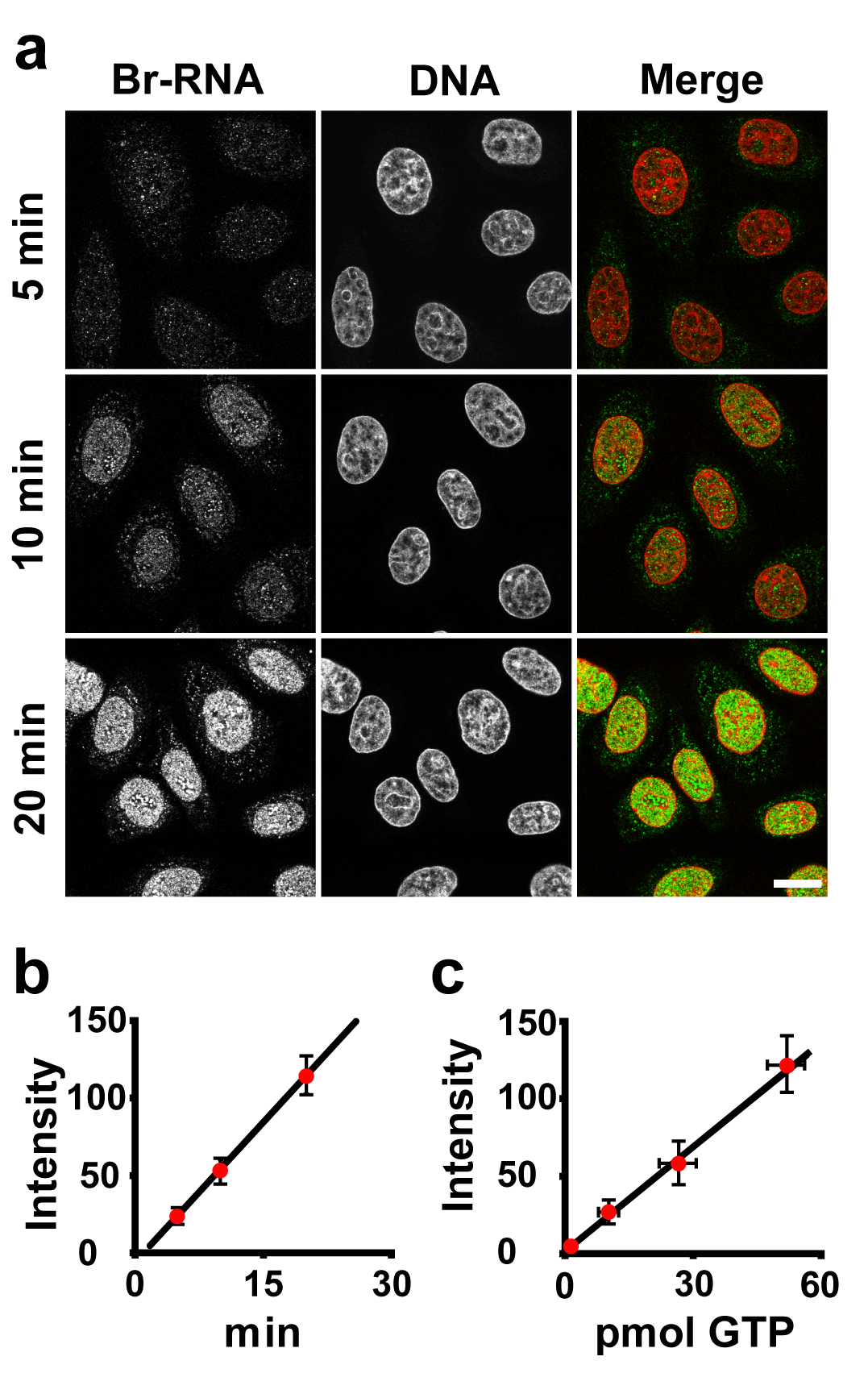

Supplement: Figure S5 — “Run on” assays. (A) Incorporation of BrUTP into the nascent transcripts of permeabilized Hela cells at three consecutive time points. After immunolabelling we observed the Br-RNA signal as small foci (left panel) distributed through the nucleoplasm (middle panel). (B) Quantitative analysis of the fluorescence intensity in the nucleoplasm of at least 200 cells like the ones shown in panel (A). The intensity of Br-RNA increases in a manner proportional to the run-on time. This graph presents data acquired with 100 µM NTP concentration. (C) Validation of the immunofluorescent approach to monitor transcription elongation. In this panel we plot the intensity of fluorescence obtained from experiments like the one shown in (B) against P32GTP incorporation into nascent RNA in cells [18], using the same incubation times. Bar = 10 µm. (1.38 MB TIF) [file pbio.1000560.s005.tif]

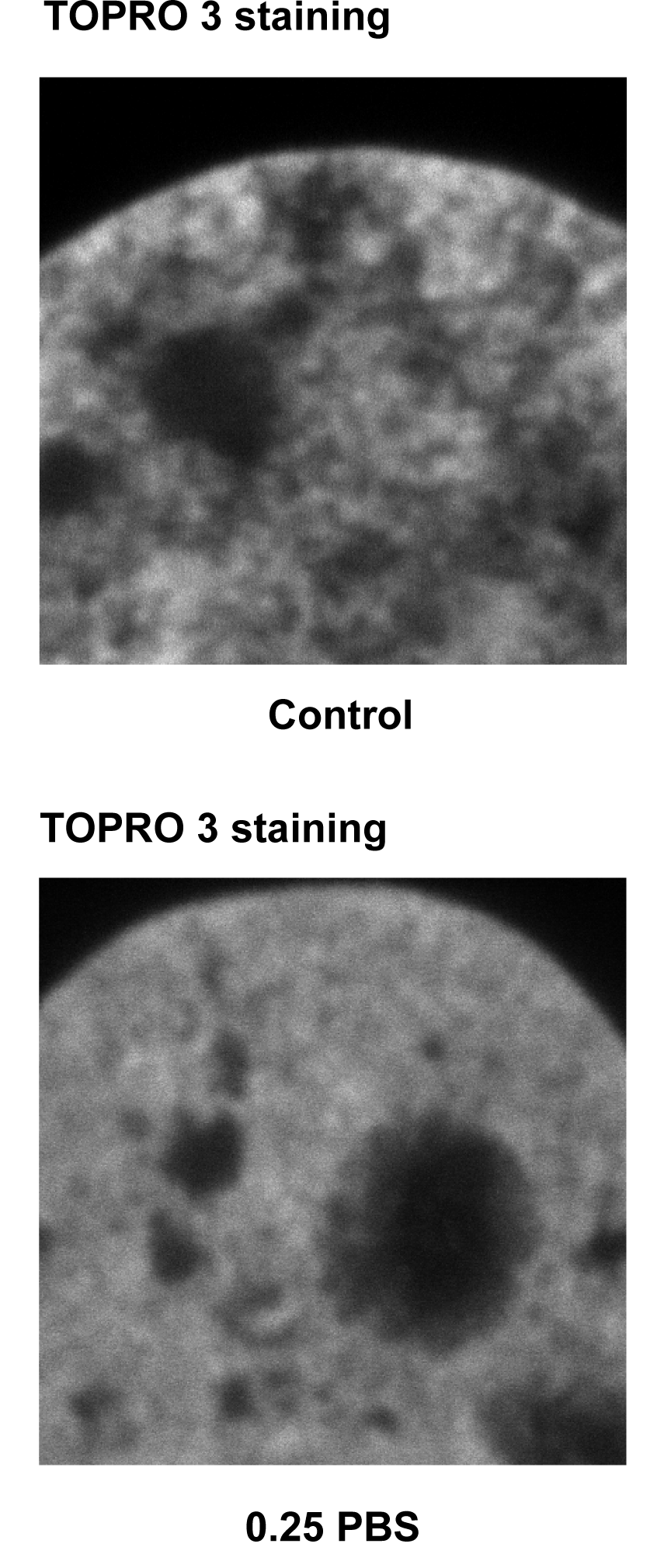

Supplement: Figure S6 — Chromatin condensation. (A) Chromatin in intact cells with clear areas of condensed and decondensed chromatin. (B) Cell nucleus after 10 min of exposure to 0.25× PBS: we can observe a progressive decondensation of chromatin (more homogeneous grey staining) with a reduced difference between dark and bright areas. (1.18 MB TIF) [file pbio.1000560.s006.tif]

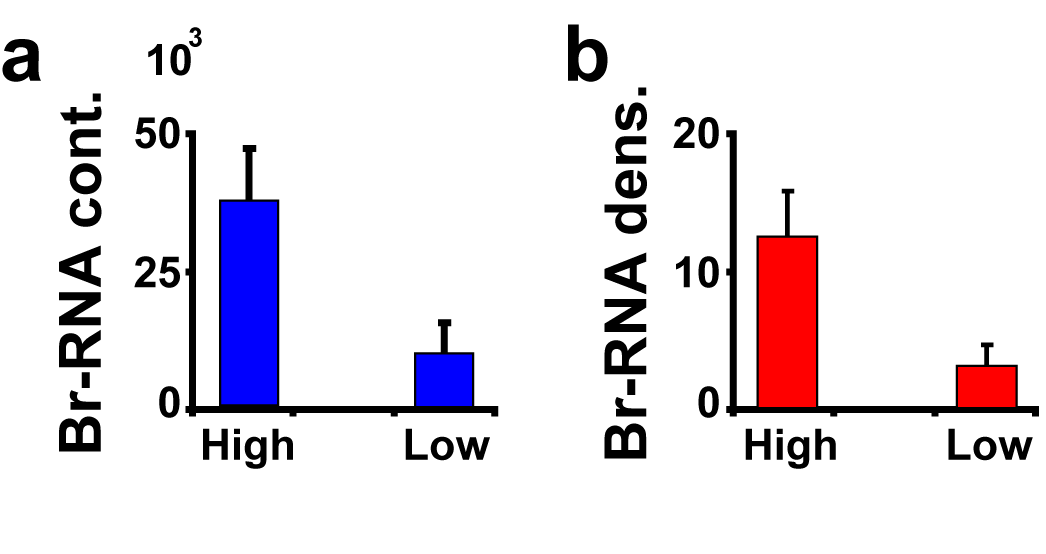

Supplement: Figure S7 — Energy and transcription elongation. (A) Energy depletion affects RNA pol II elongation. ATP was depleted by incubation of C23 cells with 10 mM sodium azide and 6 mM DG for 30 min in HBSS. This treatment reduces ATP concentration in cells by 95% (p>0.99). (B) The analysis of the half-life of the elongating form of RNA pol II shows a very strong increase: RNA pol II is almost stalled at the DNA. (0.06 MB TIF) [file pbio.1000560.s007.tif]

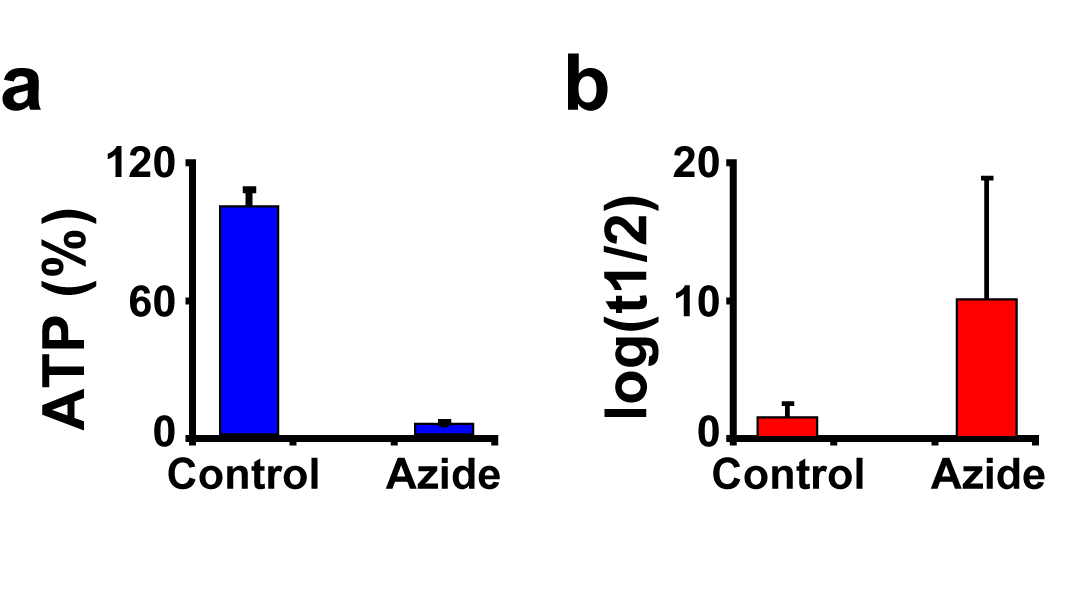

Supplement: Figure S8 — Connecting transcription rate with mitochondrial mass. For the sorted cells in Figure 3A we looked at (A) total BrU incorporation in the cells (4 h after plating, cells were incubated with BrU) (arbitrary units) and (B) the amount of BrU incorporation per unit volume of nuclear volume in populations R1 and R2 (arbitrary units). (0.22 MB TIF) [file pbio.1000560.s008.tif]

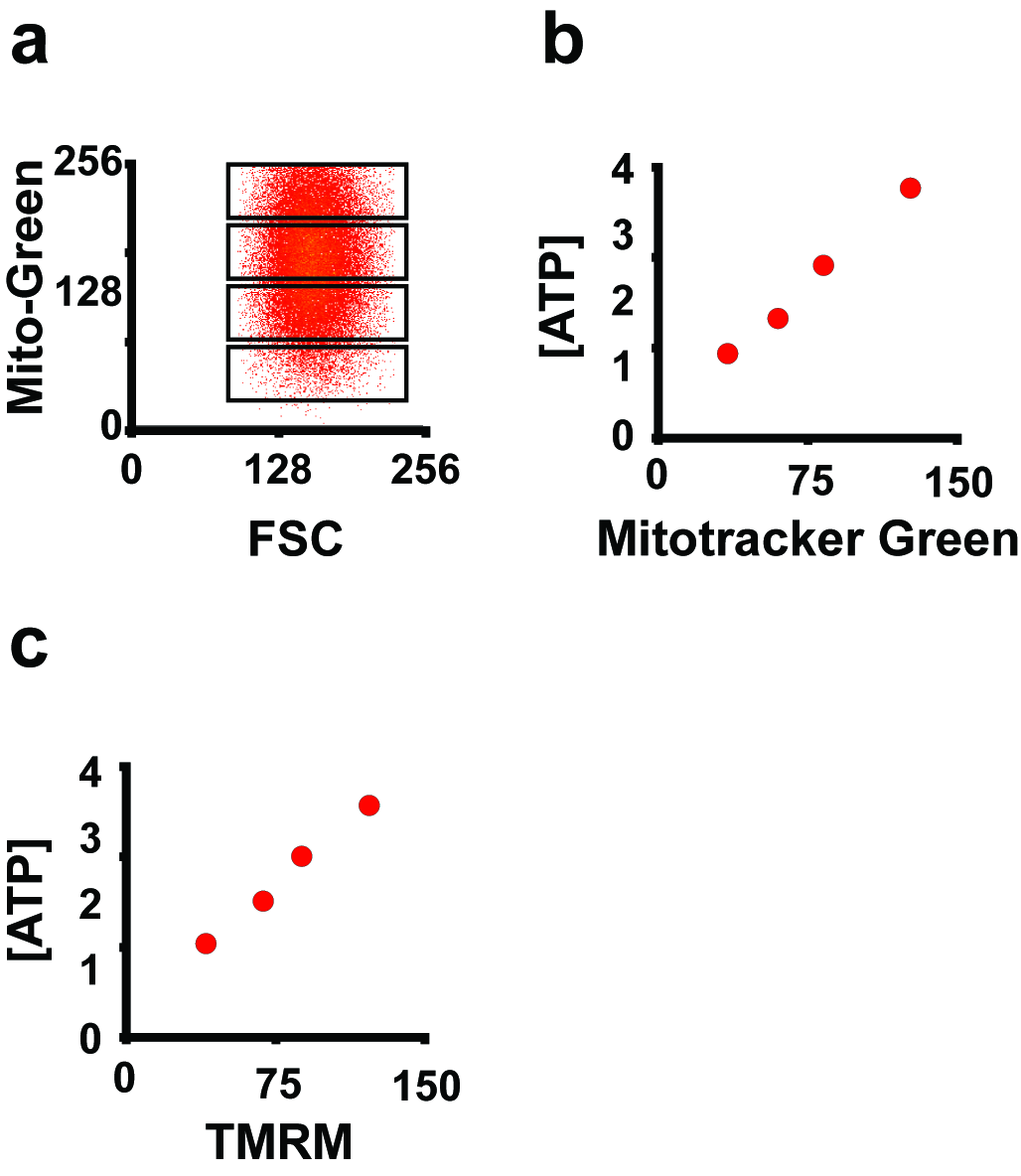

Supplement: Figure S9 — Mitochondria and energy content. (A) Cells sorted according to mitochondrial content. Four regions with different mitochondrial content were sorted (boxes). Interestingly, we found that the average volume of cells in each of these fractions was approximately constant. Making the crude assumption that the average cellular volume in all four fractions is the same, ATP per average cell in each of the four regions is an approximation for [ATP] (B) ATP content was determined in these fractions. The approximate concentration of ATP per cell increases with the mitochondrial content. (C) Analysis of approximate [ATP] in cells sorted according to TMRM content. Again, a good relation is shown. (1.11 MB TIF) [file pbio.1000560.s009.tif]

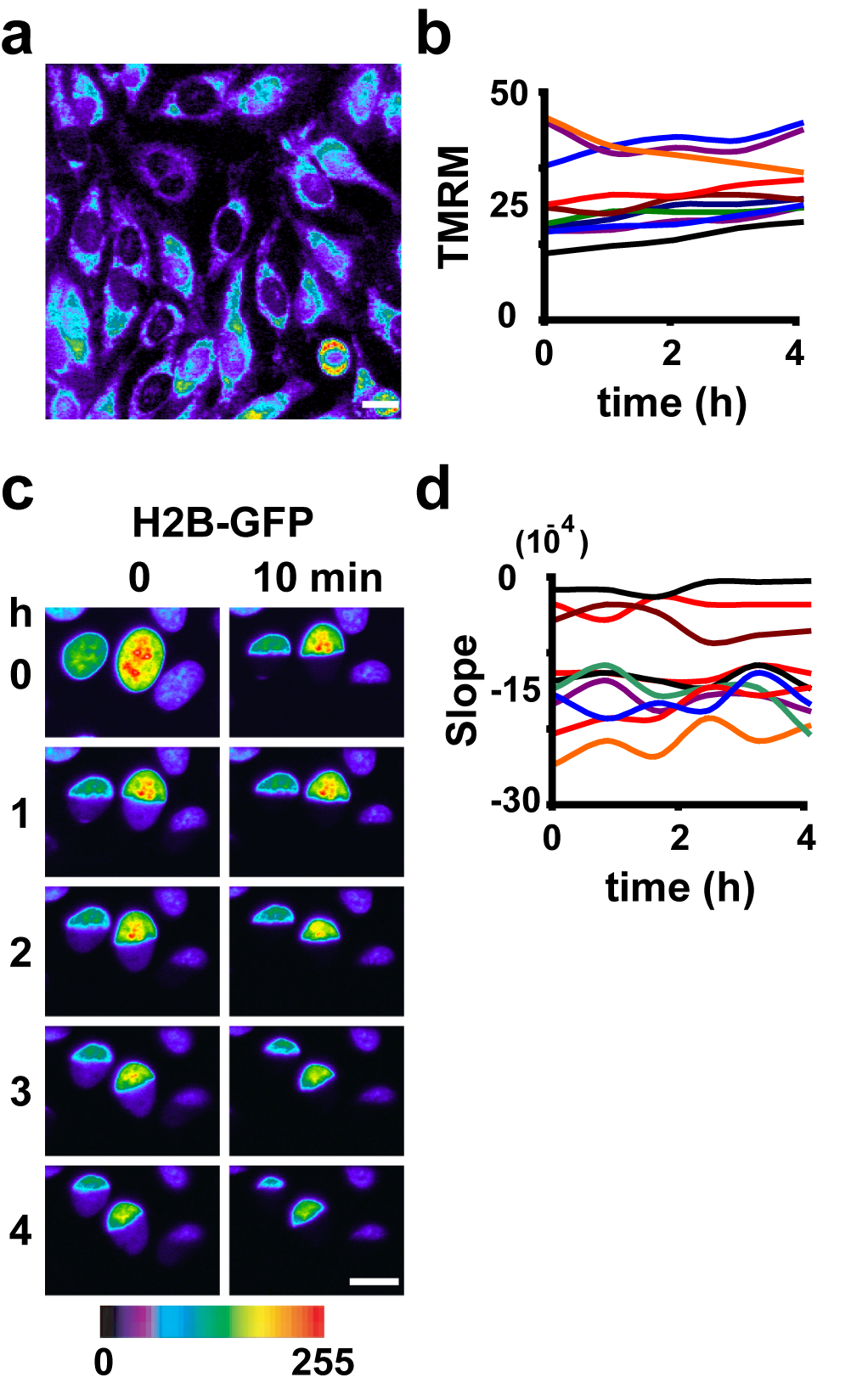

Supplement: Figure S10 — Energy potential and RNA pol II elongation are slowly varying over the time. (A) TMRM staining of Hela cells. (B) TMRM intensity fluctuation in individual cells. Images like that in (A) were taken every 20 min. (C) Hela cells expressing histone H2B–GFP were bleached for 10 min and left 50 min to recover. This bleaching cycle was repeated four times. The loss in fluorescence was measured in the unbleached part of the cell nucleus, and the faster exchanging population of histone H2B–GFP was analysed. (D) The dynamic properties of H2B–GFP do not change much over the 4 h studied, consistent with the stability in the mitochondrial potential. Bars = 10 µm. (0.96 MB TIF) [file pbio.1000560.s010.tif]

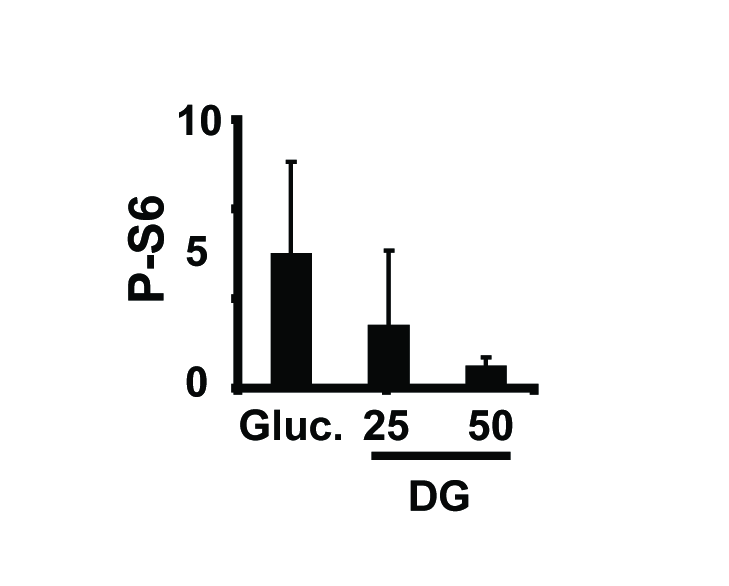

Supplement: Figure S11 — Integrated intensity of P-S6 signal in cells depleted of ATP. ATP was depleted by 12 h of incubation of cells in medium with glucose (Gluc.) and with either 25 or 50 mM DG. P-S6 appears to be a good indicator of ATP concentration in the cell. (0.60 MB TIF) [file pbio.1000560.s011.tif]

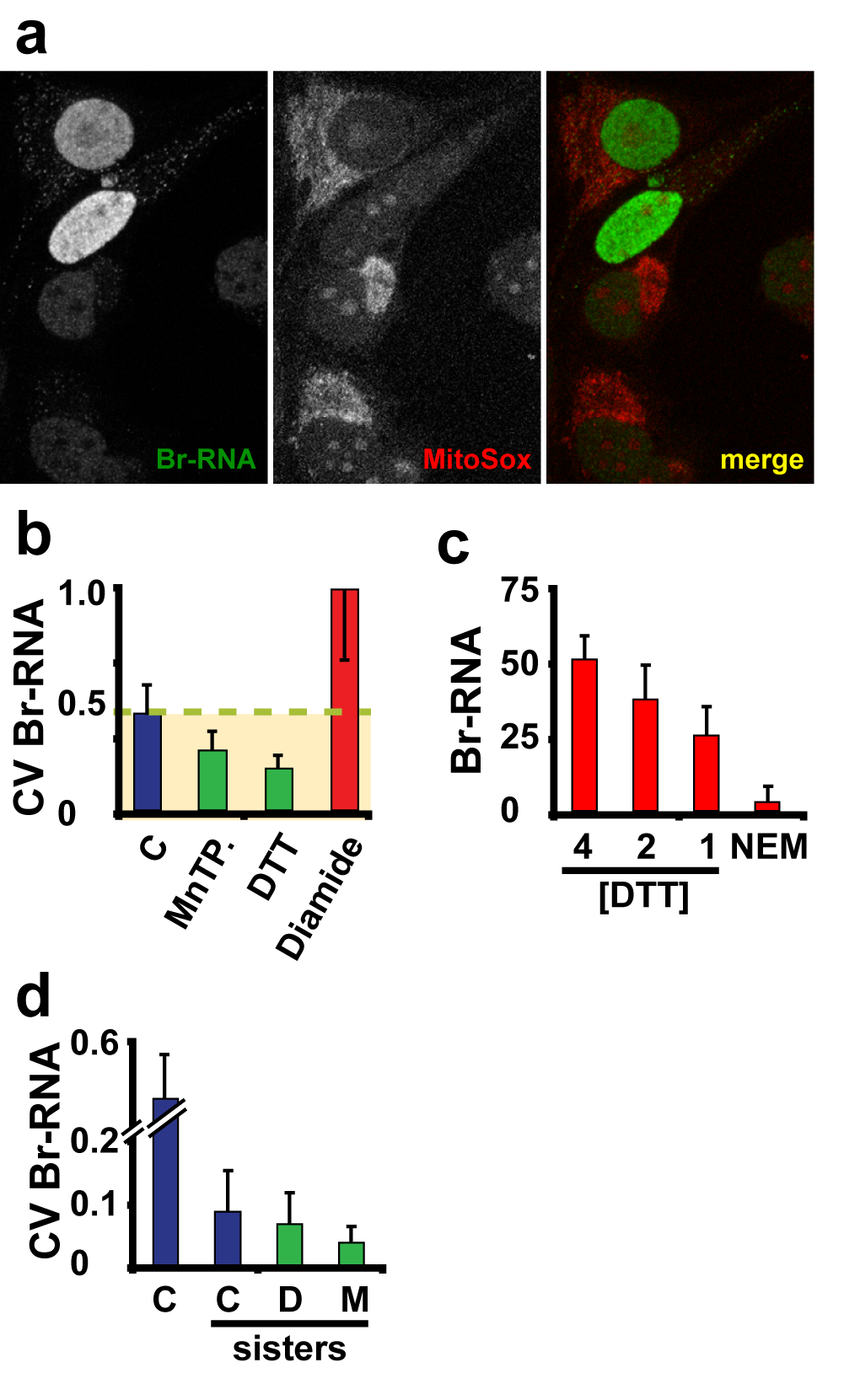

Supplement: Figure S12 — Modulating mitochondrial function modulates transcription rate. (A) The functional status of mitochondria affects transcription. Cells were incubated with MitoSox, a probe for superoxide anion detection, and then with BrU. (B) Variability in Br-RNA after different treatments. Cells were treated for 18 h with MnTMPyP (MnTP, 25 µM), DTT (125 µM), or 2 h with diamide (50 µM). Transcription variability is reduced with the antioxidants and increased with the prooxidant. (C) Effect of DTT on transcription in vitro with controlled [ATP]. DTT increases transcription rate (millimolar concentration); NEM (250 µM) reduces transcription drastically. (D) Analysis of BrU incorporation variability in the bulk population (column C), between sister cells (column C, sisters), between sister cells treated for 12 h with 125 µM DTT (column D, sisters), and between sisters cells expressing mito-YFP after normalisation by the mitochondrial content (column M, sisters). Cells were considered at all points in their cycles. The data presented are the average CV from three independent experiments. (0.75 MB TIF) [file pbio.1000560.s012.tif]

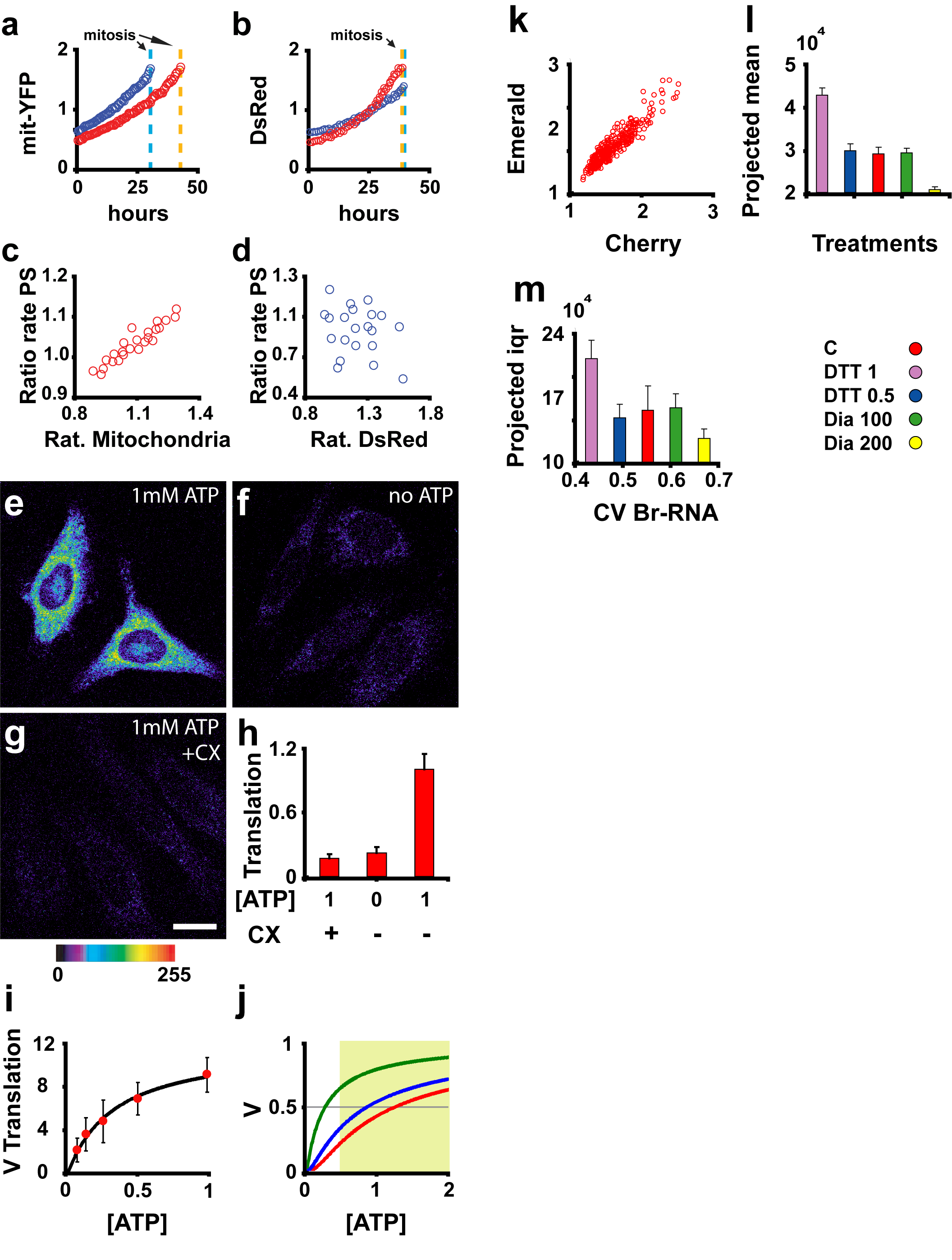

Supplement: Figure S13 — Protein synthesis and gene expression are dependent on ATP and mitochondrial content. (A) Production of mito-YFP in two daughter cells with different mitochondrial content. The mito-YFP signal grows in an exponential manner (R 2>98%). A cell with more mitochondrial content produces mito-YFP faster and completes the cell cycle in a shorter time (blue circles) than a cell with fewer mitochondria (red circles). (B) Production of DsRed protein in two daughter cells with different volume (DsRed content). The signal for DsRed grows in an exponential manner (similar to mito-YFP). (C) Relationship between the ratio of rates of protein synthesis (mito-YFP) and the mitochondrial content between daughter cells. The rate of protein synthesis was calculated as the slope of the log transformation of the mito-YFP signal growth (G). This shows that the ratio of rate of protein synthesis depends on the ratios of mitochondrial content at division. (D) Relationship between the ratio of rates of protein synthesis (DsRed) and the ratio of cellular volume between daughter cells. The ratio of rates of protein synthesis is largely independent of the cellular volume ratios. (E) Visualisation of protein synthesis by bodipy-Lys-tRNA incorporation into nascent proteins in the presence of 1 mM ATP. Bodipy is incorporated mainly into the cytoplasm of Hela cells. (F) Cells incubated with the translation cocktail lacking ATP. (G) Effect of 1 mM cycloheximide on translation. (H) Quantitative analysis of nascent translation in images like the ones displayed in (E–G) (n = 500 cells in each group). This analysis confirms that bodipy is incorporated into proteins. (I) Analysis of the velocity of translation as a function of [ATP]. This curve shows a typical Michaelis-Menten kinetic pattern. (J) Plot of model data showing the dependence on [ATP] of the different processes involved in gene expression: translation (green), transcription (blue), and transcription and translation combined (red). This analys [file pbio.1000560.s013.tif]

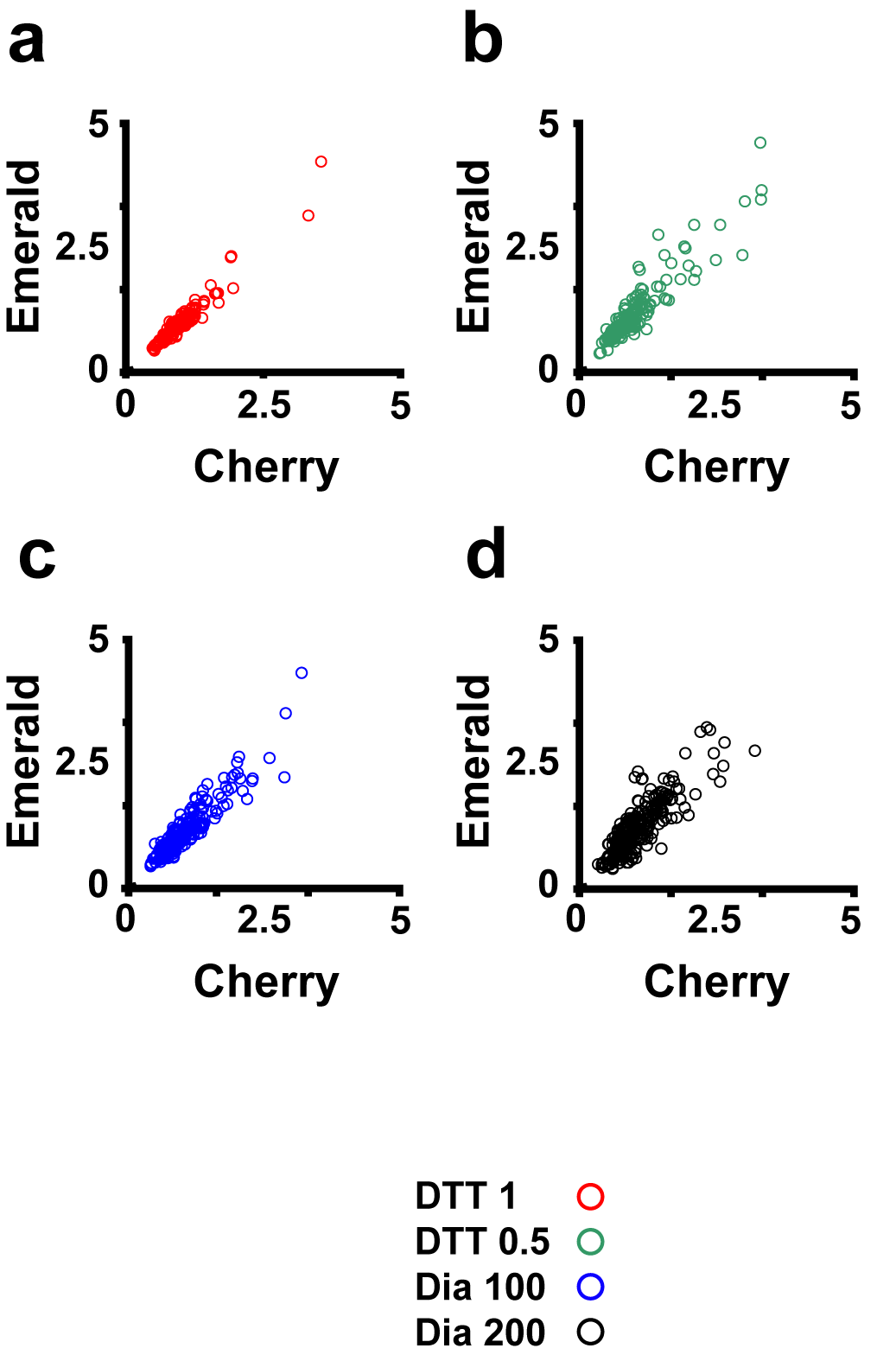

Supplement: Figure S14 — Dual reporter level fluctuations. Hela cells co-expressing Emerald and Cherry, as in Figure S13K–S13M. These panels show the analysis of intensities of both proteins in individual cells standardised by the corresponding mean value. (A) 1 mM DTT, (B) 0.5 mM DTT, (C) 100 µM diamide, (D) 200 µM diamide. (0.12 MB TIF) [file pbio.1000560.s014.tif]
