# Supplementary material for: Connecting Variability in Global Transcription Rate to Mitochondrial Variability
Source: PLoS Biol. 2010 Dec 14;8(12):e1000560. doi: 10.1371/journal.pbio.1000560 (PMC3001896; doi:10.1371/journal.pbio.1000560)
Supplement: Text S1 — Supplementary text for Figure S10. Membrane potential is slowly varying over time and so is transcription rate. (0.03 MB DOC) [file pbio.1000560.s015.doc]

**Text S1. Membrane potential is slowly varying over time and so is transcription rate**

As our measurements were for snapshots of the cellular systems, we wondered if [ATP] fluctuates over time within cells. To study the stability of intracellular [ATP] we used, as a surrogate reporter, the fluctuations in mitochondrial potential using Tetramethyl Rhodamine Methyl Ester (TMRM). After loading Hela cells with TMRM we monitored the fluctuations in fluorescence at different time scales, from seconds to hours, covering several cell cycles (Fig 4a, b). At short times (from seconds up to 10 minutes), we could not see any noticeable variation. However at longer time scales (hours), the TMRM staining in individual cells increased very slowly by about 3.8 % in an hour (movie 1), which roughly duplicates the amount of TMRM in each cell cycle. We do not have a direct measure of the cell volume variation, however, if we accept that the volume must duplicate in the course of a cell cycle then this might suggest that the concentration of ATP would change very little, if at all, in a given mammalian cell. Papers have suggested that [ATP] does not change during the cell cycle [1] but note non-trivial changes at G1-S e.g.[2]. [The above suggests that cellular ATP concentration is constant for long enough to impact on transcription. If this is the case in any given cell, the rate of transcription must be relatively stable over time. To check if this was the case we used Hela cells with histone H2B tagged with GFP and we performed five FLIP cycles on the same individual cells (Fig 4c). The analysis of the four hours FLIP time course showed that elongation is stable in individual cells (Fig 4d) with 90% of the consecutive steps changing less than 25% (note that the change in slope with time of the curve of each cell is small compared to the spread of slopes observed across the population). These results show that the speed of elongation of RNA pol II in a given cell is fluctuating relatively little over time, compared to cell-to-cell variability, probably as a consequence of stability in the energy status of the cell.

**References**

1. Chapman JD, Webb RG, Borsa J (1971) APT pool levels in synchronously growing Chinese hamster cells. J Cell Biol 49: 229-233.

2. Finkel T, Hwang PM (2009) The Krebs cycle meets the cell cycle: mitochondria and the G1-S transition. Proc Natl Acad Sci U S A 106: 11825-11826.
