# Supplementary material for: Connecting Variability in Global Transcription Rate to Mitochondrial Variability
Source: PLoS Biol. 2010 Dec 14;8(12):e1000560. doi: 10.1371/journal.pbio.1000560 (PMC3001896; doi:10.1371/journal.pbio.1000560)
Supplement: Text S2 — Supplementary text for Figure S12. Perturbing mitochondrial function perturbs transcription rate and variability. (0.03 MB DOC) [file pbio.1000560.s016.doc]

**Text S2. Perturbing mitochondrial function perturbs transcription rate and variability.**

Mitochondria are a major source of free radicals that can damage their function and can impair the generation of ATP [1]. We saw that cells generating high levels of free radicals (stained with MitoSox) have low levels of BrU incorporation. We reasoned that incubation of cells with antioxidants could improve mitochondrial function, quench free radicals, elevate [ATP], and reduce the levels of transcriptional variation. We found evidence supporting this thesis. Cells incubated for 18h with the antioxidants DTT and MnTMPyP showed a reduction in RNA pol II elongation variation (variation in BrU incorporation falls from 0.43 to 0.19 and 0.27 respectively). The opposite was also true: 2 h of incubation with the prooxidant Diamide 50 µM (which impairs mitochondrial function) resulted in an increase in transcriptional variation (from 0.43% to 0.98), this finding is consistent with increased variation in gene expression in aging due to reactive oxygen species [2]. Moreover, continuous incubation of Hela cells with 125 µM DTT reduced the interdivision time by 16%, and incubation with 50 µM Diamide increased the length of the cell cycle by 52%;.

Free radicals may affect transcriptional activity independently of ATP depletion. To isolate the contribution of redox to transcription rate we study transcription in permeabilised cells where we fixed [ATP] to 1 mM and reducing (DTT) or oxidizing agents (N-ethyl maleimide (NEM)). These experiments showed that transcription was very sensitive to the redox environment. A highly reducing cocktail (DTT) increased the degree of BrU incorporation and the opposite was obtained when NEM was present.

To help quantify the contribution of mitochondria to global transcriptional variation we studied variation in daughter cells; we reasoned that transcriptional variation should be smaller between daughter cells (since they are at a similar cell cycle stage and have similar mitochondrial content) than between pairs of cells chosen at random from the bulk population. For this analysis, individual cells expressing mito-YFP were seeded at low frequency and 24h later, when most of the cells had already divided, BrU was incorporated. Transcriptional variation in daughter cells was dramatically lower than in the bulk population (CVs of 0.08 vs. 0.47 respectively), and this variability was further reduced when daughter cells were incubated with DTT for 18 h (0.06). Moreover, we could further reduce variation when transcription in sister cells was normalized by the mitochondrial content (0.03).

**References**

1. Tiwari BS, Belenghi B, Levine A (2002) Oxidative stress increased respiration and generation of reactive oxygen species, resulting in ATP depletion, opening of mitochondrial permeability transition, and programmed cell death. Plant Physiol 128: 1271-1281.

2. Bahar R, Hartmann CH, Rodriguez KA, Denny AD, Busuttil RA, et al. (2006) Increased cell-to-cell variation in gene expression in ageing mouse heart. Nature 441: 1011-1014.
