# Supplementary material for: Connecting Variability in Global Transcription Rate to Mitochondrial Variability
Source: PLoS Biol. 2010 Dec 14;8(12):e1000560. doi: 10.1371/journal.pbio.1000560 (PMC3001896; doi:10.1371/journal.pbio.1000560)
Supplement: Text S3 — Supplementary text for Figure S13. Mitochondrial variation and translation variation. (0.03 MB DOC) [file pbio.1000560.s017.doc]

**Text S3. Mitochondrial variation and translation variation**

One might expect that the amount of mitochondria at birth affects the biosynthetic rate of the cell as well as its cell cycle duration. Evidence for this was found when we analysed the production of YFP-mito protein over time in daughter cells: cells with a higher initial mass of mitochondria appeared to translate proportionally faster (Figure 4G and 4I). On the other hand, we could not find a clear relationship between the volume of the daughter cells at birth (measured with DsRed) and the protein synthesis (Figure 4H and 4J).

The dependence of translation on [ATP] is expected because this molecule can be used to recycle GTP needed for initiation, and is needed for amino acid translocation mediated by EF2 requiring GTP hydrolysis [1]. Moreover, translation depends on the phosphorylation of the ribosomal protein S6 (PS6) by S6 kinase 1 (S6K1) which is phosphorylated by mTOR in response to [ATP] [2]. In fact, experiments of energy deprivation showed that protein synthesis (measured by PS6) decreases in a manner proportional to the reduction of Br-RNA production, which depends on [ATP] (Fig S10a-e). A simple representation of the dependence of gene expression on [ATP] is as the product of the two processes (transcription and translation). This gives a sigmoidal curve with higher Km than each one of the former processes individually (Km = 1.26 mM) and higher dynamic range for changes in [ATP] (Fig S13 j). According to this model, gene expression is highly sensitive to [ATP]; if we compare two cells with different ATP content (0.5 mM and 2 mM), the speeds of protein synthesis, transcription and gene expression are 1.3, 2.1 and 2.75 times higher, respectively. This gives [ATP] a major role as a controller of gene expression. One obvious conclusion from this work is that the level of two proteins expressed in a single cell should be positively correlated (extrinsic variability). We find that modulating the mitochondrial function, and so the transcription rate, perturbs both mean protein levels and the spread of observed reporter-pair co-expression (Figure S13 k-m).

**References**

1. Alberts B (2008) Molecular biology of the cell. New York: Garland Science.

2. Dennis PB, Jaeschke A, Saitoh M, Fowler B, Kozma SC, et al. (2001) Mammalian TOR: a homeostatic ATP sensor. Science 294: 1102-1105.
